# Aptamer-based inhibition of MNK1 reduces pancreatic ductal adenocarcinoma growth by targeting cancer stem cells

**DOI:** 10.1186/s12929-026-01275-6

**Published:** 2026-07-06

**Authors:** Alberto Pérez-Ruiz, Laura Ruiz-Cañas, Sandra Batres, Raquel Ferreras-Martín, Celia Pinto-Díez, Balbino Yagüe, Maria Isabel Pérez-Morgado, Silvia Sacristán, Ignacio Ruz-Caracuel, Sonia Camaño Páez, Isabel Sanchez-Perez, Víctor M. González, Sonia Alcalá, María Elena Martín, Bruno Sainz

**Affiliations:** 1 Aptamer Group, Department of Biochemistry-Research, Instituto Ramón y Cajal de Investigación Sanitaria (IRYCIS)-Hospital Universitario Ramón y Cajal, 28034 Madrid, Spain; 2 Department of Cancer, Biomedical Research Institute (IIBm) Sols-Morreale CSIC-UAM, 28029 Madrid, Spain; 3 Biomarkers and Personalized Approach to Cancer Group (BIOPAC), IRYCIS, Area 3 Cancer, 28034 Madrid, Spain; 4 Biobank Hospital Ramón y Cajal-IRYCIS, Spanish National Biobanks Network (ISCIII Biobank Register No. B. 0000678), IRYCIS, 28034 Madrid, Spain; 5 Aptus Biotech SL, Av. Cardenal Herrera Oria 298, 28035 Madrid, Spain; 6 Pathology Department, Hospital Universitario Ramón y Cajal, 28034 Madrid, Spain; 7 Universidad Autónoma de Madrid, School of Medicine, Department of Biochemistry, 28029, Madrid, Spain; 8 Molecular Pathology of Cancer Group, IRYCIS, Area 3 Cancer, 28034 Madrid, Spain; 9 Centro de Investigación Biomédica en Red, Área Cáncer, CIBERONC, Instituto de Salud Carlos III, 28029 Madrid, Spain; 10 Centro de Investigación Biomédica en Red, Área Enfermedades Raras, CIBERER, Instituto de Salud Carlos III, 28029 Madrid, Spain

**Keywords:** Aptamer, Cancer stem cells, Pancreatic ductal adenocarcinoma, MNK1

## Abstract

**Background:**

Pancreatic ductal adenocarcinoma (PDAC) remains one of the deadliest cancers due to late diagnosis, early metastasis and resistance to therapy. Cancer stem cells (CSCs) have been implicated in PDAC aggressiveness and treatment failure. MAP kinase-interacting kinase 1 (MNK1) is overexpressed in PDAC and plays a critical role in tumor progression and CSC maintenance. Here, we show the potential of apMNKQ2, a DNA aptamer targeting MNK1, to therapeutically target CSCs and reduce PDAC tumor burden in patient-derived xenografts (PDXs).

**Methods:**

PDX cell lines and in vivo mouse models were used to assess the effects of apMNKQ2 on cell viability, apoptosis, cell cycle progression, migration, epithelial-to-mesenchymal transition (EMT) and CSC properties. Functional CSC targeting was validated through clonogenic and self-renewal assays as well as extreme limiting dilution analysis. Systemic administration of free apMNKQ2 was tested for biodistribution, pharmacokinetics, toxicity, and antitumor efficacy at escalating doses.

**Results:**

apMNKQ2 downregulated MNK1 and anti-apoptotic proteins (MCL1, XIAP), impaired cell proliferation, induced apoptosis, and disrupted cell cycle progression in PDX PDAC cells. Importantly, apMNKQ2 also inhibited migration, mesenchymal properties and angiogenesis in vitro, and lung colonization in vivo. Notably, apMNKQ2 strongly targeted PDAC CSCs, reducing CD24, CD133, CXCR4 and ALDH expression, clonogenicity and in vivo tumor initiation over 600-fold. Free apMNKQ2 (without transfection agents) entered PDAC cells efficiently, retaining anti-CSC activity. Systemic delivery of free apMNKQ2 accumulated in tumors, was well tolerated up to 400 mg/kg and showed no toxicity. Importantly, 10 mg/kg of apMNKQ2 produced strong antitumor effects in PDX models. Increasing the dose 20-fold enhanced tumor uptake but not efficacy, suggesting a therapeutic plateau at 10 mg/kg.

**Conclusions:**

MNK1 plays a central role in PDAC progression and CSC maintenance. apMNKQ2 is a potent anti-MNK1 DNA aptamer with robust preclinical activity, including CSC-targeting and anti-invasive effects. Its low toxicity, systemic bioavailability, and efficacy at low doses support further development as a novel therapeutic strategy for PDAC.

**Supplementary Information:**

The online version contains supplementary material available at 10.1186/s12929-026-01275-6.

## Background

MAP kinase-interacting kinases (MNKs) are kinase enzymes involved in various cellular processes, such as regulation of protein synthesis, cell growth and survival [8]. MNKs bind to and are substrates for mitogen-activated extracellular signal-regulated protein kinases (ERK)1/2 and stress-activated MAP kinases (p38 MAPKs), which are crucial for cellular responses to stress, growth factors and oncogenic signals [25, 53]. In humans, two genes (MKNK1 and MKNK2) encode MNKs, which produce two isoforms by alternative splicing: MNK1a/MNK1b and MNK2a/MNK2b [44, 47]. Their best-characterized substrate is the translation initiation factor eIF4E [53], but other substrates have also been described, including heterogeneous nuclear ribonucleoprotein [7], polypyrimidine stretch-binding protein-associated splicing factor [6], phospholipase A [31] and Sprouty [15]. MNK isoforms are overexpressed in different types of cancer, such as glioblastoma, lymphoma, breast, lung, prostate and ovarian cancer and hepatocellular carcinoma, and their high expression levels are associated with poor prognosis [28, 30, 46, 52, 55]. In addition, MNKs play a role in oncogenesis and metastasis by regulating the levels and activity of proteins involved in apoptosis (MCL1, XIAP, survivin), proliferation (Cyclins, CDKs, c-myc, β-catenin), migration and epithelial-mesenchymal transition (ZEB1, Snail, MMPs, NDRG1) [44].

MNK1 overexpression increases proliferation, migration and invasion in hepatocellular carcinoma cell lines [52], while MNK1 knockdown causes decreased viability of prostate cancer cells [49] and increased sensitivity to rapamycin in glioblastoma [28]. MNK1 signaling also promotes the progression of primary breast cancer from ductal carcinoma in situ to invasive ductal breast carcinoma [29]. Moreover, MNK1 is highly expressed in pancreatic acinar cells in mice and is activated after induction of pancreatitis, an important risk factor for the development of pancreatic ductal adenocarcinoma (PDAC) [10]. Thus, since MNK1 plays a role in cancer cell growth and survival, it has been explored as a potential therapeutic target, as a means of suppressing tumor growth and/or sensitizing cancer cells to chemotherapy or targeted therapies.

Aptamers are short, single-stranded nucleic acids (DNA or RNA) capable of acquiring a tertiary structure that binds specifically to a target molecule. These molecules have garnered significant attention in the biomedical field for their ability to bind specifically to a wide range of molecular targets, including proteins, cells, and small metabolites. The development of aptamers leverages a technique known as SELEX (Systematic Evolution of Ligands by Exponential Enrichment), which allows for the selection of aptamers with high affinity and specificity for their intended targets [18, 51]. Aptamers have emerged as promising therapeutic agents in cancer therapy because of their ability to specifically bind to cancer-related molecules, such as cell surface markers or proteins that play key roles in tumor progression [16, 57]. Although there are a large number of aptamers in clinical trials as therapeutics in different diseases, only two aptamers have entered clinical trials for the treatment of cancer: AS1411 (NCT01034410, NCT00881244, NCT00512083), which targets nucleolin [3], a multifunctional protein that is overexpressed in cancer [5] and NOX‐A12 (NCT04121455, NCT04901741), an RNA aptamer that inhibits tumor growth by binding to CXCL‐12 [36].

In addition to cancer, aptamers have demonstrated their potential in applications ranging from immune response modulation to the treatment of infectious diseases. The therapeutic use of aptamers presents several advantages over conventional antibodies, such as their smaller size, which enhances tissue penetration, and their inherent stability, which reduces the need for stringent storage and handling conditions. Furthermore, unlike antibodies, aptamers can be synthesized chemically, minimizing the risk of variability often associated with biological production. Moreover, their ability to deliver therapeutic payloads, such as drugs or imaging agents, also makes them versatile tools in personalized medicine. So far, only two aptamers have been approved by the FDA for therapeutic use in humans: Macugen^®^, approved for the treatment of age-related macular degeneration [27], and Izervay, for geographic atrophy (GA) secondary to age-related macular degeneration [37]. However, numerous aptamers have entered clinical trials for different diseases [1, 38, 42]. Our group previously developed ApTOLL, a TLR4 antagonist aptamer and potential drug for the treatment of ischemic stroke [21], which has already passed phases 1 and 2a clinical trials [34, 35].

As aptamer technology advances and our understanding of their role in molecular biology deepens, their therapeutic potential is expected to expand considerably. However, several challenges still need to be addressed that will significantly transform the landscape of molecular therapy and pharmacology, mainstreaming the use of aptamers for the treatment of diverse pathologies. These include, the stability of aptamers in the human body, which can limit their efficacy due to nuclease degradation; delivery to the target tissue or cell (although aptamers are small and potentially easier to administer than antibodies, the development of efficient delivery systems (such as nanoparticles or liposomes) to ensure the aptamers reach the tumor is crucial for success); and toxicity and safety (although aptamers present lower toxicity than traditional chemotherapies, their safety must be thoroughly evaluated).

In our laboratory, we have developed the aptamer apMNKQ2 by optimizing the parental aptamer apMNK2F, selected against MNK1b [26]. The apMNKQ2 aptamer is a 29-nucleotide DNA aptamer, which due to the presence of two G-quadruplex structures, is highly resistant to nucleases. Likewise, we have previously shown its potential antitumoral effect in tumor lines and xenograft models of both breast [45] and lung cancer [9]. In this study, we extensively characterize the antitumor properties of the MNK1-targeting aptamer apMNKQ2, demonstrating its capacity to inhibit key oncogenic processes in PDAC. PDAC remains one of the deadliest solid tumors, with a five-year survival rate of less than 12% and a median overall survival of under one year for patients with advanced disease [20]. Its poor prognosis stems from late diagnosis, early metastatic spread, and an intrinsic resistance to most conventional and targeted therapies. Increasing evidence suggests that these hallmarks are driven by a small but highly tumorigenic and stem-like subpopulation of cancer stem cells (CSCs), which possess self-renewal, differentiation, metastatic and tumor-initiating capacities [33]. CSCs are believed to contribute not only to tumor maintenance and progression but also to therapy resistance and relapse, making them a critical therapeutic target. Given the absence of clinically effective CSC-targeting agents, novel strategies capable of eliminating this subpopulation are urgently needed. In this study, we present a comprehensive preclinical evaluation of apMNKQ2, a chemically stable, G-quadruplex-forming DNA aptamer designed to inhibit MNK1, a kinase we show herein to be implicated in PDAC progression and CSC maintenance. We assessed the ability of apMNKQ2 to reduce tumor growth and lung colonization, target the CSC compartment in vitro and in vivo, as well as its pharmacokinetics, biodistribution, safety, and efficacy as a systemically administered therapeutic. Through this approach, we aimed to establish MNK1 inhibition via DNA aptamer technology as a novel and promising strategy against PDAC, particularly through its ability to target the PDAC CSC compartment.

## Methods

### Cell culture and transfection

PDAC PDXs were obtained from Dr. Manuel Hidalgo under a Material Transfer Agreement with the Spanish National Cancer Centre (CNIO), Madrid, Spain (Reference no. I409181220BSMH). All PDAC PDX tumors contained G12D mutations in KRAS as determined by PCR sequencing as described in [14]. To establish low-passage primary PDX-derived in vitro cultures, PDXs tumors were minced, enzymatically digested with collagenase (Stem Cell Technologies) for 60 min at 37 °C, clarified via multiple sequential rounds of filter purification with 100 µm and 40 µm Fisherbrand^™^ Sterile Cell Strainers (FisherScientific, Cat no. 11517532 and 11587522), and after centrifugation for 5 min at 1800 rpm, the cell pellets were resuspended and cultured in RPMI (Hyclone, Cat no. SH30096.01) supplemented with 10% FBS (Gibco, Cat no. A5256701), 50 units/ml penicillin/streptomycin and fungizone (Gibco, Cat no. 15240062). PDX-derived cultures are referred to by a random number designation (e.g., Panc185, PancA6L, Panc215, or Panc354). Primary cultures were tested for Mycoplasma at least every 4 weeks.

PDX-derived Panc cell lines were treated with p29 ssDNA (GCGGTCGACTTAAATGTCCATCTCAAACT) or apMNKQ2 (TGGGGTGGGCGGGCGGGGGTGGGGGTGGT) aptamers using the transfection Lipofectamine^™^ 2000 system (Invitrogen, Cat no. 11668019) following the manufacturer’s instructions for siRNA transfection. The DNA-lipofectamine complex was added to the cells previously washed twice with antibiotic-free medium to prevent interference with the lipofectamine. The cells were incubated at 37 °C for 5 h, after which the reagent was removed, and complete medium was added. For free apMNKQ2 treatments, apMNKQ2 was added to the culture media at the concentrations indicated. To calculate transfection efficiency, apMNKQ2 conjugated with Alexa488 or FITC was added to the cells and the number of fluorophore-positive cells was analyzed either by fluorescence microscopy (Olympus IX70) or by flow cytometry (Attune™ NxT Acoustic Cytometer, Thermo Fisher Scientific).

### Cell viability assay

The four PDX Panc cell lines were seeded at 1.5 × 10^4^ cells/well in 96-well plates. After 16–24 h, p29 ssDNA or apMNKQ2 were transfected at the concentrations indicated in the figure legends using Lipofectamine^™^ 2000. After 48 h, the medium was removed and 100 µL of MTT (1 mg/mL in culture medium) (Invitrogen, Cat No. M6494) was added to each well, and the plates were incubated for 1–4.5 h at 37ºC. Then, 100 µL/well of lysis buffer (10% SDS and 10 mM HCl) was added and, after 24 h of incubation, the absorbance was read at 540 nm in a SpectraFluor microplate reader (TECAN, Switzerland). The results are expressed as the percentage of viability relative to control.

### Proliferation assays

PDX-derived Panc cell lines were collected 24 h post transfection, counted by trypan blue exclusion assay (Biorad, Cat no. 1450003) and seeded at 250 cells/well in 6-well plates. Approximately 10 days later, cells were fixed, stained for 30 min with 0.02% Giemsa (Sigma, Cat no. WG16-500), and counted with a magnifying glass (× 1.75) (Bel-Art Scienceware, Wayne, New Jersey, USA).

### Scratch wound-healing assays

The PDX Panc354 cells were plated in 24-well plates at 5 × 10^4^ cells/well. When cells reached 100% confluence, they were transfected with p29 ssDNA or apMNKQ2 at 125 nM and incubated in serum-free medium. After 16 h of serum deprivation, cells were treated with 1 μM mitomycin C (Sigma, Cat no. 10107409001) for 2 h. Subsequently, the monolayer was scraped with a P-200 pipette tip, washed with medium to remove floating cells and photographed with an Olympus IX70 with a 2 × objective in phase contrast (time 0). The media was refreshed with 2.5% FBS containing RPMI and pictures were taken after 15 h to avoid complete wound closure in the control condition. The wound area was quantified at both times using the ImageJ program with the macro Wound Healing Tool. The results are expressed as the percentage of wound closure compared to the control.

### Zymograms

Zymograms were performed to analyze MMP9 and MMP2 activity in the medium of PDX-derived Panc cell lines treated or untreated with apMNKQ2. After transfection, cells were incubated in serum-free medium for 20 h and the medium from each condition was centrifuged for 5 min at 210 g at 4 °C to remove cell debris. The same volume of medium was then transferred to a 3 kDa Amicon^®^ centrifuge filter (Merck, Cat no. UFC500396) and centrifuged for 30 min at 14,000 g at 4 °C to concentrate the samples. Twenty µl of medium were mixed with loading buffer without beta-mercaptoethanol and resolved on a 7.5% SDS-PAGE polyacrylamide gel co-polymerized with 1 mg/mL gelatin. Gels were incubated for 30 min in 2.5% Triton X-100 (Sigma, Cat no. T8787) at room temperature and overnight at 37ºC in developing buffer (Tris 50 mM pH 7.5, NaCl 200 mM, CaCl_2_ 5 mM, Tween 0.02%). Gels were stained with Coomassie blue for 15 min and unstained until MMP activity appeared as clear bands.

### Adult human dermal microvascular endothelial cells (HMVEC-d Ad) tubule formation assay

Tubule formation assay for HMVEC-d Ad (Lonza, Cat no. K3CC-2543) was performed as previously described [23]. Briefly, a 48-well plate was polymerized with 100 μl of Growth Factor Reduced Matrigel (Corning, Cat no. CLS356231) for 30 min at 37 ℃. HMVEC-d Ad (3 × 10^4^) were incubated in 100 μl of conditioned medium (CM) diluted 1:4 in EGM-2 Endothelial Cell Growth Basal Medium (Lonza, Cat no. H3CC-3202). The CM was obtained from Panc215 or Panc354 control cells or cells transfected with p29 control or apMNKQ2 aptamer during 24 h at IC50 concentration. After 12 h, five images per well and condition were taken using an Olympus IX70 microscope with a 2 × objective in phase contrast. The capillary tubes were quantified and the results were expressed as the percentage of vessel numbers relative to control.

### Western blot analysis

To obtain protein lysates, cells were washed once with cold buffer A [20 mM Tris–HCl pH 7.6, 1 mM dithiothreitol (DTT), 1 mM ethylenediaminetetraacetic acid (EDTA), 1 mM phenylmethylsulfonyl fluoride (PMSF), 1 mM benzamidine, 10 mM sodium molybdate, 10 mM sodium β-glycerophosphate, 1 mM sodium orthovanadate, 120 mM potassium chloride (KCl), 10 µg/mL antipain, 1 µg/mL pepstatin A and leupeptin], and mechanically harvested and centrifuged at 400 g for 5 min. Cell pellet was lysed in the same buffer containing 1% Triton X-100 (volume ratio 1:2) and centrifuged at 12,000 g for 10 min. Subsequently, protein concentration was determined using a BCA Kit (ThermoFisher Scientific, Cat no. 23227) and supernatants were aliquoted and stored at − 80 °C until used.

Twenty micrograms of protein were resolved by sodium dodecyl sulfate–polyacrylamide gel electrophoresis (SDS-PAGE) and transferred to PVDF membranes (GE Healthcare). Membranes were sequentially blocked with PBS containing 5% milk (w/v), incubated with a 1:500–1:1000 dilution of different antibodies overnight at 4 °C, washed three times with PBS containing 0.05% Tween20 (v/v), incubated with horseradish peroxidase-conjugated goat anti-rabbit or goat anti-mouse antibody (GE Healthcare), and washed again to remove unbound antibody. Bound antibody complexes were detected with Clarity Western ECL Substrate (BioRad, Cat no. 170–5060). The β-actin antibody (Sigma, Missouri, USA) was used as a control to monitor the homogeneity of loading. The intensity of the bands was recorded using a Chemidoc imaging system (BioRad) and quantified using Image Lab software (BioRad). Antibodies used in this work can be found in Supplementary Table S1. Uncropped western blot images for all figures are found in Supplementary Figures S9-13.

### RNA-seq and analysis

RNA was isolated from PDX Panc354 and Panc215 cells, both control and treated with apMNKQ2, in triplicate, using the Total RNA purification kit (NORGEN, Cat no. SKU 17200) following the manufacturer's protocol. RNA concentration and quality was analyzed using a TapeStation bioanalyzer (Agilent). Libraries were prepared using the Stranded mRNA Prep, Ligation Kit (Illumina). The resulting purified cDNA library was sequenced with the Novaseq 6000. Quality control, adapter trimming, and alignment of RNA-seq data were performed using the nf-core/rnaseq pipeline (10.5281/zenodo.1400710). FASTQ data files generated have been deposited in the European Nucleotide Archive (ENA) with accession number ID: PRJEB94044.

### Gene expression datasets and GSEA analyses

Gene set enrichment analysis (GSEA) was performed using annotations from Hallmark and additional gene sets from the Molecular Signature Database (MSigDB) (www.broadinstitute.org/gsea/index.jsp), as detailed in Supplementary Table S2. The GSEA module of the Genepattern suite from the Broad Institute was used, with 1000 permutations and FDR < 25% was considered statistically significant. The dataset from Bailey et al. was included in a supplementary figure of their published work [2]. The samples included in the top and bottom quartiles of *MKNK1* expression were compared in GSEA, using the Hallmark geneset database.

### RNA extraction

RNA from blood (50 µL) and tumor tissues (50–100 mg) were lysed and homogenized in NucleoZOL (Macherey–Nagel, Cat no. 2301–001) following the manufacturer’s protocol for isolation of small and large RNA in two separate fractions. Both RNA fractions were resuspended in 50 μL of RNase-free water, quantified and stored at − 80° C. The small RNA fraction was used for apMNKQ2 quantification.

### Quantification of apMNKQ2 by qPCR

To quantify apMNKQ2 in blood and tumors, the purified small RNA fraction was treated with RNAse A (ThermoFisher Scientific, Cat no. EN0201) (0.2 µg/µL 30 min at 37 °C, 10 min at 65ºC) to remove RNA that could interfere in the quantification of apMNKQ2. We have developed a method in which apMNKQ2 acts as a primer on a template called QR short (5′-ACACCAGTCTTCATCCGCTCGTATTTAGACCAGTCTTCATCCGCACCACCCCCACCCCC-3′). The aptamer apMNKQ2 that is present in this fraction is elongated generating a 76-nucleotide template that can then be amplified with the primers named F3c (5′ACACCAGTC TTCATCCGC3′) and QF (5′-TGGGGTGGGCGGGC-3′), being the amplification proportional to the amount of apMNKQ2 present in the sample. The qPCR was performed using AceQ qPCR SYBR^®^ Green Master Mix (Vazyme, Cat no. Q111-02) according to the manufacturer's instructions in a StepOne Plus Real-Time PCR system (Applied Biosystems, Waltham, MA, USA). The aptamer was quantified using a standard curve (100–0.1 fmol).

### Flow cytometry and cell sorting

For flow cytometric analyses cell suspensions were blocked with Flebogamma (Grifols) for 15 min at 4 °C. For the detection of CD24, CD133 or CXCR4 cell surface marker expression, cells were incubated with a 1:5 dilution of a mouse anti-human CD24 PE (BD Cat no. 555428), a 1:50 dilution of a mouse anti-human CD133/1 Vio Bright R667 Ab (Miltenyi Cat no. 130–111–756) or a 1:50 dilution of a mouse anti-human CXCR4 (CD184) PE Ab (Miltenyi Cat no. 130–117–354). For ALDH activity, the Aldefluor^™^ Kit (STEMCELL Technologies Cat. no. 01700) was used, with 33 µM DEAB (4-Diethylaminobenzaldehyde, inhibitor of ALDH) for ALDH + cell gating. For all assays, 2 μg/ml DAPI (4’,6-diamidino-2-phenylindole, Sigma, Cat no. D9542-5MG) was used to exclude dead cells. Cells were resuspended in Flow buffer [1X PBS; 3% FBS (v/v); 3 mM EDTA (v/v)] before analysis. All samples were analyzed using a 4-laser Attune™ NxT Acoustic Cytometer (Thermo Fisher Scientific). Data analysis was performed using FlowJo software (Tree Star Inc., Ashland, OR).

### Histopathology

Specimens were fixed in 10% buffered formalin and embedded in paraffin or OCT. For histopathological analyses, tissues were serially sectioned, and every 4th section was stained with hematoxylin and eosin (H&E). Representative sections were chosen for the grading and enumeration of lesions and quantification of tissue damage. For PCNA or XIAP staining, tumor sections were deparaffinized and rehydrated by a graded ethanol series and washed with PBS. Antigen retrieval was achieved by heat treatment in a pressure cooker for 2 min in 10 mM citrate buffer (pH 6.5). Next, endogenous peroxidase was blocked, and the sections were incubated with anti-PCNA or anti-XIAP 1:1000 overnight at room temperature. For PCNA, sections were incubated with anti-mouse-biotin 1:500 (DAKO, Cat no. E0354) for 45 min followed by ABC-HRP kit (Vectastain, Cat no. PK-6100) for 30 min at room temperature. For XIAP, sections were incubated with Master Polymer Plus Detection System (Peroxidase) (Master Diagnostica, Cat no. MAD-000237-QK) for 30 min at room temperature. Finally, sections were developed with the Inmunoperoxidase DAB kit (Master Diagnostica) according to the manufacturer’s instructions. Sections were counterstained with hematoxylin.

Cytokeratin 19 (CK19) and anti-human Nucleoli staining was performed in an automated immunostaining platform (Discovery ULTRA, Ventana-Roche and Autostainer Link 48, Dako), including deparaffinization and re-hydrated. Antigen retrieval was first performed with the appropriate method (CC1m, Roche and Low pH buffer, Dako, Agilent) and endogenous peroxidase was blocked (hydrogen peroxide at 3%). Then, slides were incubated with a mouse monoclonal anti-CK19 (RCK108, prediluted; Dako-Agilent, IR0615) or mouse monoclonal anti-Nucleoli (NM95, 1/18,000, Abcam, 190710), followed by an anti-mouse-HRP 1:500 (Abcam, ab133469) antibody. Immunohistochemical reaction was developed using 30-diaminobenzidine tetrahydrochloride (ChromoMap DAB, Ventana, Roche). Finally, nuclei were counterstained with Mayer’s hematoxylin and all the slides were dehydrated, cleared and mounted with a permanent mounting medium for microscopic evaluation. Positive control sections known to be primary antibody positive were included for each staining run.

A Motic EasyScan One was used to acquire whole images and images were scaled and annotated with Motic DS Assistant software. Positive cells were analyzed using ImageJ software.

### Colony assay

For colony formation assays, 500 cells were seeded in 24-well plates. apMNKQ2 was added or transfected (as previously described) 24 h post seeding at the indicated concentration(s). Cells were cultured in RPMI 1640 containing 10% FBS at 37 °C, 5% CO2. After 10–12 days, cells were fixed with PFA 4% (Paraformaldehyde, 16% w/v aq. soln., methanol free, Alfa Aesar™, Cat no. 11400580) for 10 min, washed with PBS and stained with Crystal violet (Sigma, Cat no. C3886-100G) for 1 h. Wells were digitalized and colonies/total area were quantified by lysing stained colonies in 1X PBS with 1%SDS followed by colorimetric absorbance analysis using a Synergy™ HT Multi-Mode Microplate Reader (BioTek, Winooski, Vermont, USA). For free apMNKQ2 studies, cells were either pre-treated with increasing doses of apMNKQ2 for 24 h prior to establishing colonies (i.e., pre), or treated when colonies were established (i.e., post).

### Spheroids culture

For spheroid formation assays, pancreatic CSC spheroids were generated by culturing PDX-derived Panc cells (2000 cells/ml) transfected with p29 or apMNKQ2 in ultra-low attachment plates (Corning) using serum-free DMEM/F12 (Invitrogen, Cat no. 21331046) supplemented with B27 1:50 (Invitrogen, Cat no. 17504044), 20 ng/ml bFGF (PAN-Biotech, Sigma, Cat no. GF446-10UG), L-Glutamine (Invitrogen, Cat no. 25030081), 50 units/ml penicillin/streptomycin (Invitrogen, Cat no. 11548876) and fungizone (Invitrogen, Cat no. 15290018). Spheroid formation was quantified using a Casy TTC cell counter (OMNI Life Science GmbH & Co KG, Bremen, Germany). Counts > 40 µm were considered spheroids. For free apMNKQ2 studies, cells were either pre-treated with increasing doses of apMNKQ2 for 24 h prior to establishing spheres (i.e., pre), or treated when spheres were established (i.e., post).

### In vivo *assays*

For assessing migratory and lung colonization capacity, 5 × 10^5^ Panc354 cells, control transfected or transfected with 100 µM of apMNKQ2, were resuspended in 100 μl physiological saline (0,9% NaCl) and slowly injected into the tail vein of 10-week-old NOD.Cg-Prkdc scid/Rj mice (Janvier). Mice were sacrificed 120 days post-tail vein injection. For the extreme limiting dilution assays (ELDA), serial dilutions of Panc354 cells, control transfected or transfected with 100 µM of apMNKQ2, were resuspended in 1:4 primary culture medium:Matrigel™ in a final volume of 50 µl and injected into the flanks (right and left) of athymic Rj:ATHYM-Foxn1 nu mice (Janvier). Animals were examined twice a week for tumor formation up to 4 months, and were euthanized whenever 100% tumor take was achieved in one of the two groups and/or when a humane endpoint sacrifice for one of the two groups was reached (i.e., the diameter of the tumor reached 1cm^3^).

For assessing the tumorigenic potential of PDX cell lines transfected with apMNKQ2, 5 × 10^5^ Panc215 or Panc354 cells, control transfected or transfected with 100 nM apMNKQ2, were resuspended in 50 μl Matrigel^™^ and slowly injected into the pancreas of 10-week-old NOD.Cg-Prkdc scid/Rj mice (Janvier). Mice were sacrificed at the indicated weeks post injection. For the aforementioned experiments, after euthanasia, full necropsy was performed and tumors/organs were extracted, photographed and weighed. Tumors were cut into pieces for RNA isolation, protein extraction or flow cytometric analysis, and/or fixed in 4% PFA overnight at 4 °C for histology analysis.

To track the fluorescence emitted by apMNKQ2 in real time post injection, 6–7-week-old CD1swiss mice (Janvier) were administered a single dose of 1.7 mg/kg of apMNKQ2 either intravenously via retro-orbital injection (r.o.) or intraperitoneally (i.p.) in a total volume of 100 µl of selection buffer (20 mM Tris–HCl pH 7.4, 150 mM NaCl, 1 mM MgCl2, 5 mM KCl) or conjugated with in vivo-jetPEI^®^ (Polypus, Cat no. 201-50G) at a N/P ratio of 6. Mice were anesthetized using isoflurane and fluorescence was visualized using the IVIS-Lumina II (Caliper Life Sciences, Hopkinton, MA, USA) and analyzed by the software Living image 3.2 (Perkin Elmer, Waltham, MA, USA) at the indicated times post injection.

For biodistribution and toxicity assays, 6–7-week-old CD1swiss mice (Janvier) were administered apMNKQ2 at the doses indicated (e.g., 10, 100, 200 or 400 mg/kg) via i.p. or r.o. Mice were continuously monitored for acute toxicity. At 4 h post injection with 10 mg/kg of apMNKQ2, a group of CD1swiss mice was euthanized and kidneys, liver, brain, lungs and pancreas were collected and snap frozen at − 80 °C for subsequent RNA extraction. Blood was collected in EDTA tubes (Aquisel, Cat no. 107545), centrifuged at 2000 × g for 10 min at 4 °C and plasma was frozen at − 80 °C for subsequent quantification of apMNKQ2 by qPCR, as described above.

For ADME-Tox assays, 6 to 8-week-old female C57Bl/6 J mice (Charles River) were administered with apMNKQ2 at 10, 100, 200 or 400 mg/kg via i.p. injection, daily for 4 weeks. Mice were continuously monitored for acute toxicity. Mice were euthanized and brain, intestine, heart, lung, liver, spleen, kidneys and pancreas were harvested, fixed in 4% PFA and subsequently embedded in paraffin for histological analyses. Blood was collected in EDTA tubes (Aquisel, Cat no. 107545). Fifteen µl of whole blood was used for hematocrit analysis (Element HT5, Veterinary Hematology Analyzer, scil animal care company GmbH, Madrid, Spain). For pharmacokinetic (PK) assays, 6- to 8-week-old female C57Bl/6 J mice (Charles River) were administered with apMNKQ2 at 10 mg/kg via i.p. injection and blood was collected from individual mice at 5, 10, 15 and 30 min and 1, 2, 4, 8 and 24 h via cardiac puncture. Blood was centrifuged at 2000 × g for 10 min at 4 °C and plasma was frozen at −80 °C for subsequent quantification of apMNKQ2 by qPCR, as described above.

For PDX in vivo treatment experiments, tumors were initially established by subcutaneous implantation of tumor pieces of the indicated PDXs into 6- to 8-week-old athymic Rj:ATHYM-Foxn1 nu mice (Janvier). Three weeks later, tumors were measured to ensure similar initial volumes of 125–150 mm^3^. Mice were weighed and randomized into groups. Ten mg/kg or 200 mg/kg of apMNKQ2 were tested: 1) via retro-orbital injection (100 µl, daily) – r.o. or 2) i.p (100 µl, daily). Tumor volumes were determined twice per week by caliper measurements. At the time of euthanasia, mice were weighed, blood was collected in EDTA tubes (Aquisel, Cat no. 107545) and tumors/organs were excised and weighed, photographed, frozen at − 80 °C for subsequent qPCR analysis and/or fixed in 4% PFA and processed for histological analysis.

All in vivo procedures in mice were conducted in accordance with protocols approved by the Use Committee for Animal Care from the Universidad Autónoma de Madrid (UAM) (Ref# CEI-25–587) and the Comunidad de Madrid (PROEX 294/19, 205.23). Mice were housed according to institutional guidelines and all experimental procedures were performed in compliance with the institutional guidelines for the welfare of experimental animals and in accordance with the guidelines for Ethical Conduct in the Care and Use of Animals as stated in The International Guiding Principles for Biomedical Research involving Animals, developed by the Council for International Organizations of Medical Sciences (CIOMS).

### Statistical analyses

Results are presented as means ± standard error of the mean (SEM) unless stated otherwise. Pair-wise multiple comparisons were performed with one-way ANOVA (two-sided) with Bonferroni or Dunnett adjustment, as indicated in the figure legends. Student’s t-test was used to determine differences between means of groups. P-values < 0.05 were considered statistically significant. All analyses were performed using GraphPad Prism version 6.0c (San Diego, California, USA).

## Results

### apMNKQ2 inhibits the viability and proliferation of PDX-derived PDAC cell lines

**Fig. 1:**
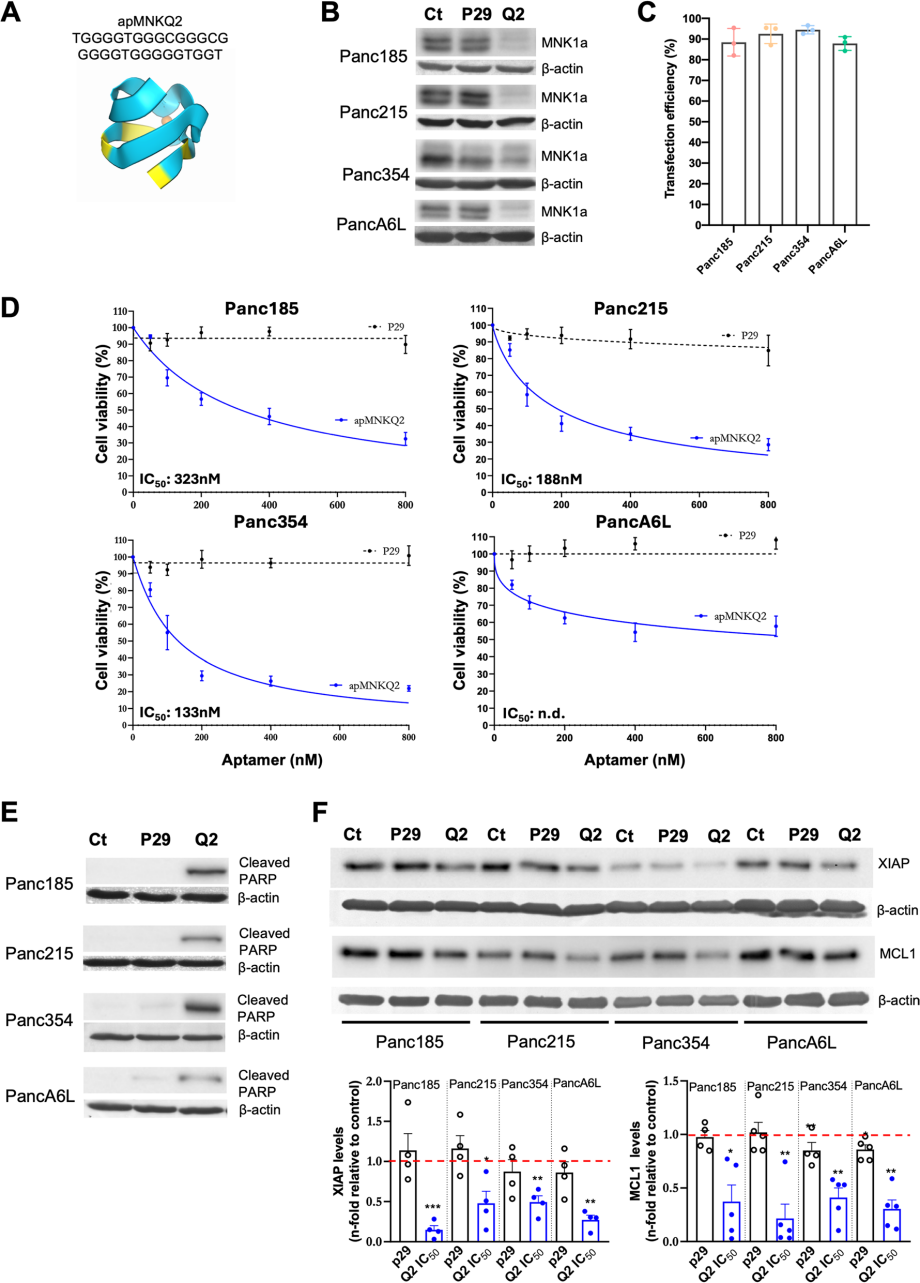
apMNKQ2 inhibits the viability and proliferation of PDX-derived PDAC cell lines. **A** Sequence of apMNKQ2 (top) and 3D structures predicted by AlphaFold 3. **B** WB analysis of MNK1 protein levels in the four PDX-derived cell lines 24 h after p29 (control) or apMNKQ2 aptamer transfection (200 nM). β-actin is included as a loading control. A representative blot of four independent experiments is shown. **C** Transfection efficiency of apMNKQ2 in the four PDX-derived cell lines was measured by fluorescence microscopy. Alexa488-apMNKQ2 was transfected at 200 nM during 16 h and the number of cells positive for aptamer relative to total cells (stained with Hoechst) were quantified. The graph shows the mean ± SEM of 3 experiments. **D** Four PDX-derived cells were transfected with p29 (control) or apMNKQ2 aptamer at different concentrations (0–800 nM). After 48 h, cell viability was measured by MTT activity. The graphs represent the means ± SEM of 4 independent experiments. **E** WB analysis of cleaved PARP in the four PDX-derived cell lines 24 h after p29 (control) or apMNKQ2 aptamer transfection at IC50 concentration. β-actin is included as a loading control. Top, a representative blot of four independent experiments of cleaved-PARP protein levels; bottom, quantification of the levels of PARP protein. The graph represents the means ± SEM of 4 independent experiments. **F** WB analysis of XIAP and MCL1 protein levels in the four PDX-derived cell lines 24 h after p29 (control) or apMNKQ2 aptamer transfection at IC50 concentration. β-actin is included as a loading control. The graphs represent the means ± SEM of 4–5 independent experiments. Dashed red line represents the 1.0-fold change of the control. A representative blot is shown in the top of the figure. *p < 0.05; **p < 0.01; ***p < 0.001 relative to control; as determined by one-sample t test

The objective of this study was to evaluate the antitumor potential of the 29 nucleotide aptamer apMNKQ2 (Fig. 1A) in pancreatic cancer. For this purpose, we first assessed the expression of MNK1 by western blot analysis (WB) in four PDX-derived PDAC cell lines, confirming that PDAC-derived cells express MNK1, and that apMNKQ2 significantly reduces its expression (Figs. 1B and S1A). Next, to assess the therapeutic range of apMNKQ2, all PDX-derived PDAC cell lines were transfected with increasing concentrations of the aptamer. All four PDX-derived PDAC cell lines showed similar transfection efficiency (Fig. 1C). Cell viability assays revealed that PDX Panc354 was the most sensitive (IC_50_ = 133 nM), followed by PDX Panc215 (IC_50_ = 188 nM) and PDX Panc185 (IC_50_ = 323 nM), whereas PDX PancA6L exhibited resistance, with no measurable IC_50_ (Fig. 1D). The negative control p29, a random sequence of 29 nucleotides (GCGGTCGACTTAAATGTCCATCTCAAACT), did not affect MNK1 protein levels (Fig. 1B) nor did it induce nonspecific toxicity (Fig. 1D).

To determine whether the observed reduction in cell viability resulted from apoptosis, we evaluated cleaved-PARP, a key apoptotic marker, by western blot 24 h after aptamer transfection. An increase in the caspase-3-specific PARP fragment was detected in all four PDX-derived cell lines after aptamer transfection (Fig. 1E) in parallel with a decrease in the levels of total PARP (Figure S1B). The control P29 did not induce an increase in the caspase-3-specific PARP fragment **(**Fig. 1E**).** Additionally, we examined the levels of XIAP and MCL1, two anti-apoptotic proteins regulated by MNK1 [19, 46, 54]. Treatment with apMNKQ2 significantly reduced XIAP and MCL1 expression in all tested cell lines, confirming its pro-apoptotic effect (Fig. 1F). The inhibitory effects of apMNKQ2 also extended to cell proliferation, as demonstrated in a Giemsa-based proliferation assay. At IC25 and IC50 concentrations specific to each cell line, apMNKQ2 significantly impeded cell proliferation (Figure S1C).

In our previous reports, we demonstrated that apMNKQ2 also affected cell cycle and migration/invasion [9, 45]. Along these lines, we analyzed the cell cycle profile of PDAC cell lines treated with apMNKQ2 at IC25 and IC50 concentrations. Among the four cell lines studied, Panc354 exhibited a statistically significant increase in the proportion of cells in the G2/M phase relative to control, while Panc185, Panc215, and PancA6L showed a trend toward G2/M accumulation that did not reach statistical significance (Figure S1D). To elucidate the molecular basis of this cell cycle perturbation, we evaluated the phosphorylation status of CDK2^Tyr15^ and histone H3^Ser10^, established markers of G1/S and mitotic arrest, respectively. ApMNKQ2 treatment led to a marked reduction in CDK2^Tyr15^ phosphorylation, indicative of an absence of G1/S checkpoint activation. Conversely, histone H3^Ser10^ phosphorylation was increased, suggesting mitotic arrest (Figure S1E). Since the Cyclin B/CDK1 complex governs G2/M transition and mitotic progression, we further assessed the impact of apMNKQ2 on these proteins. Both CDK1 and Cyclin B levels were significantly reduced following apMNKQ2 treatment, suggesting that cells fail to progress beyond mitosis (Figure S1F). These findings implicate a translational blockade during mitosis that disrupts mitotic progression, potentially through impaired synthesis of key mitotic regulators.

### apMNKQ2 inhibits the migratory capacity and invasive capacity of PDAC cells

**Fig. 2:**
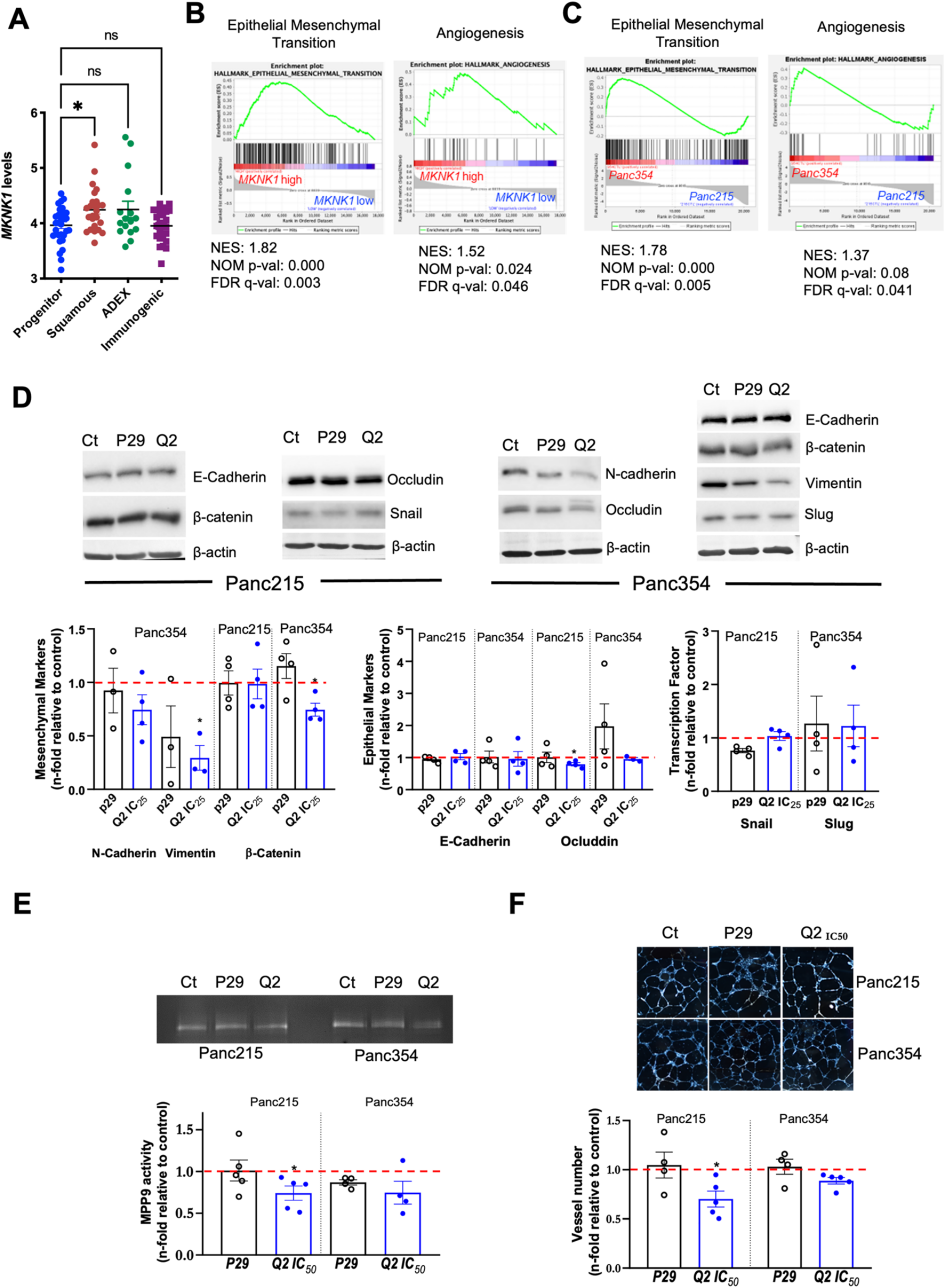
apMNKQ2 inhibits the migratory and invasive capacity of PDAC cells.** A** Fragments Per Kilobase of exon per Million mapped reads (FPKM) of *MKNK1* levels in the Bailey dataset series containing 96 PDAC patients, classified as progenitor, squamous, aberrantly differentiated endocrine exocrine (ADEX) or immunogenic. *p < 0.05 determined by One-way ANOVA with Dunnett's multiple comparison test. **B** Gene sets enriched in the transcriptional profile of tumors belonging to the top *MKNK1* high expression group, compared with the bottom expression group in the Bailey data series. Shown are the NES (normalized enrichment score) values for each pathway using the Hallmark genesets, meeting the significance criteria: nominal p value of < 0.05, FDR < 25%. **C** Gene sets enriched in the transcriptional profile of Panc354 cells compared with Panc215 cells. Shown are the NES (normalized enrichment score) values for each pathway using the Hallmark genesets, meeting the significance criteria: nominal p value of < 0.05, FDR < 25%. **D** WB analysis of EMT and cell membrane protein levels in Panc215 and Panc354 cell lines 24 h after p29 (control) or apMNKQ2 aptamer transfection at IC25 concentration. β-actin is included as a loading control. The graphs represent the means ± SEM of 4 independent experiments. Representative blots are shown in the top of the figure. **E** MMP9 activity in Panc215 and Panc354 cell lines 24 h after p29 (control) or apMNKQ2 aptamer transfection at IC50 concentration was measured by zymography. The graphs represent the means ± SEM of 5 independent experiments. Dashed red line represents the 1.0-fold change of the control. *p < 0.05 relative to control; as determined by one-sample t test. A representative zymogram is shown in the top of the figure. **F** The effect of apMNKQ2 on angiogenesis was analyzed using the HMVEC-dAd cell Tube Formation Assay. Conditioned medium from p29- or apMNKQ2-transfected Panc215 and Panc354 cells was added to HMVEC-d Ad cells seeded in Matrigel, and the formed tubules were photographed 12 h later. The graph represents the means ± SEM of 4–5 independent experiments. All dashed red line represents the 1.0-fold change of the control. Representative microphotographs are shown in the top of the figure. *p < 0.05 relative to control; as determined by one-sample t test

To translate our findings to the patient setting, we probed the Bailey et al., dataset containing 96 PDAC patients, classified as progenitor, squamous, aberrantly differentiated endocrine exocrine (ADEX) or immunogenic [2]. *MKNK1* mRNA levels were significantly higher in the squamous subtype compared to the progenitor subtype (Fig. 2A). The squamous subtype (or basal-like subtype) is correlated with significantly worse disease outcomes, with nearly half the survival rate compared to the average of the other three subtypes [12, 13, 56], and functional studies have demonstrated that induction of mesenchymal- and epithelial-to-mesenchymal transition (EMT)-associated genes correlates with the aggressive squamous/basal-like PDAC [41]. Indeed, classifying patients based on *MKNK1* levels as high or low, and performing Gene Set Enrichment Analysis (GSEA) on the top and bottom quartiles, we observed that patients with higher *MKNK1* levels were enriched in genes associated with EMT (Fig. 2B), indicating that *MKNK1* may promote a more invasive PDAC phenotype. To confirm these results, RNAseq analysis was performed on PDX Panc354 cells with high MNK1 levels and PDX Panc215 cells with lower MNK1 levels (Figure S2A) followed by GSEA. The results obtained showed that PDX Panc cells with high MNK1 levels (Panc354) were enriched in genes associated with EMT (Fig. 2C). Indeed, Panc354 cells had higher expression levels of mesenchymal markers such as N-cadherin, Vimentin and Slug and lower expression levels of epithelial markers such as Occludin, β-catenin and Claudin compared to Panc215 cells with lower MNK1 levels (Figure S2B).

To determine the effect of apMNKQ2 on mesenchymal- and EMT-associated markers/factors, we analyzed associated proteins in both PDAC cell lines treated with apMNKQ2 and observed that MNK inhibition negatively affected the protein levels of the mesenchymal markers N-cadherin, Vimentin and β-Catenin in Panc354 cells both at IC25 (Fig. 2D) and IC50 (Figure S2C) concentrations of the aptamer. The aptamer apMNKQ2 also inhibited migration of Panc354 cells (Figure S2D), indicating an inhibition in migration in these MNK1high expressing cells. Non-effect of apMNKQ2 on the levels of EMT key transcription factors was observed in both cell lines, both at IC25 (Fig. 2D) and IC50 (Figure S2C) concentrations of the aptamer (data not shown), suggesting that apMNKQ2 modulates mesenchymal markers rather than EMT factors. Additionally, zymogram analysis showed that apMNKQ2 treatment led to a marked reduction in MMP9 secretion (Fig. 2E) with no significant effect on MMP2 release (data not shown). We also observed that patients and PDX Panc cells with high levels of *MKNK1* showed a significant enrichment in genes associated with angiogenesis (Fig. 2B, C), a process involved in tumor development and progression and in metastasis [22]. To experimentally address this observation, conditioned media from control or P29- or apMNKQ2-transfected PDAC cells were tested for their capacity to induce HUVEC cell tube/vessel formation. In line with our results, apMNKQ2 reduced the capacity of Panc215 and Panc354 to induce vessels in HUVEC cells (Fig. 2F).

**Fig. 3:**
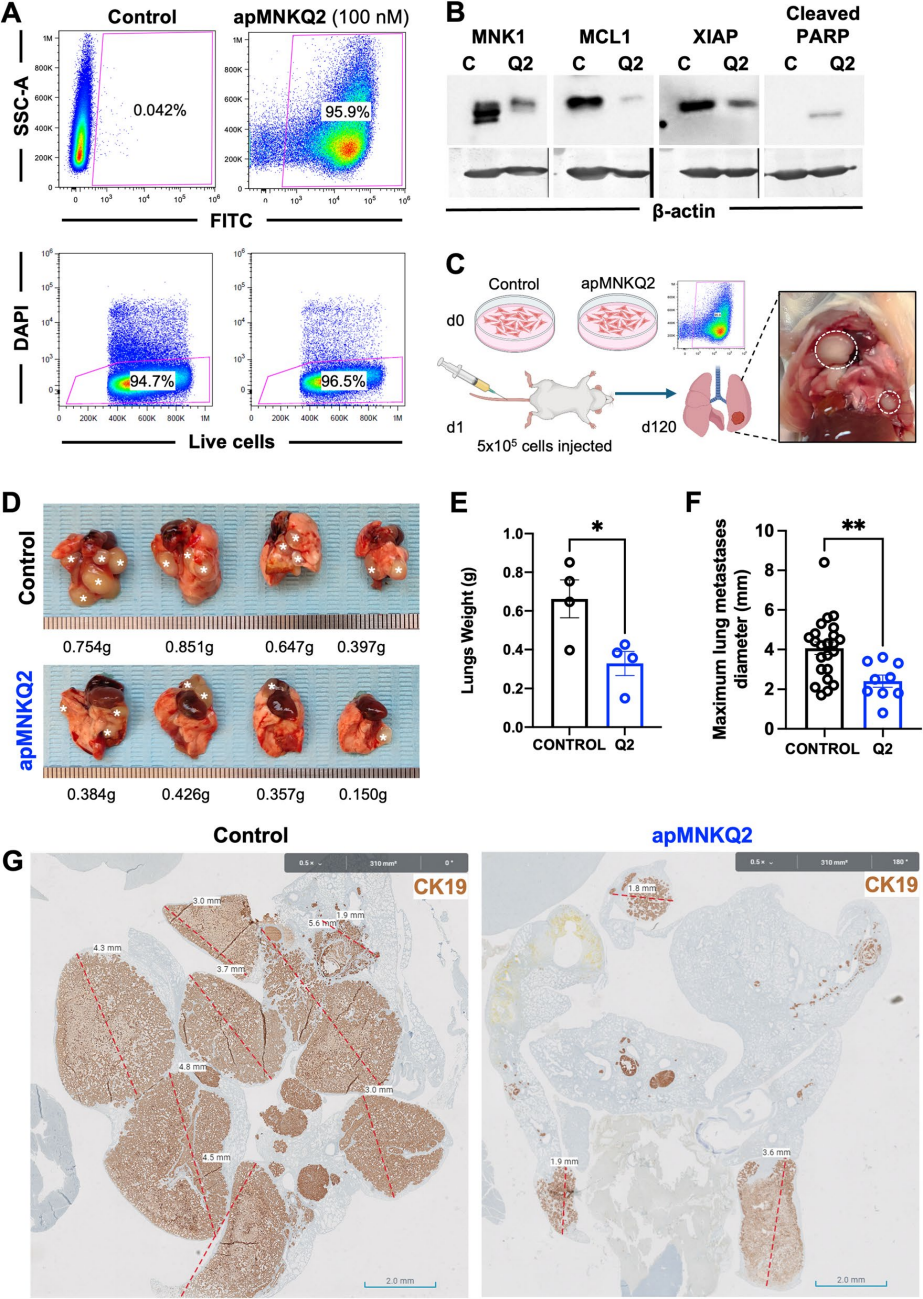
apMNKQ2 inhibits the migratory and colonization capacity of PDAC cells.** A** Representative dot plots of the percentage of FITC-positive (top) or DAPI-negative (bottom) Panc354 cells 24 h post transfection with control or apMNKQ2-FITC (100 nM). **B** WB analysis of MNK1, MCL1, XIAP or cleaved PARP protein levels in control- (C) or apMNKQ2-FITC (Q2)-transfected cells. β-actin is included as a loading control. **C** Schematic of the transfection and post tail vein injection lung colonization study performed on NOD.SCID mice. **D** Photographs of the lungs and heart extracted from NOD.SCID mice 120 days post tail vein-injection with 5 × 10^5^ control- or apMNKQ2-FITC-transfected cells. The weight of each lung is shown below. * represents macro metastases. **E** Mean lung weight (g) ± SEM from D. *p < 0.05 relative to control; as determined by one-sample t test. **F** Mean ± SEM of maximum diameter of lung metastases detected by CK19 immunohistochemistry. **p < 0.01 relative to control; as determined by one-sample t test. **G** Representative micrographs of CK19-stained lungs from D

In light of these observations, we tested the in vivo capacity of control or apMNKQ2-transfected PDAC Panc354 cells to successfully migrate in vivo and initiate lung colonization in NOD.SCID mice following tail vein injection. To this end, Panc354 cells (the PDX cell line with the highest metastatic capacity) were transfected, using Lipofectamine 2000, with 100 nM apMNKQ2 (Q2) for 24 h, revealing a high transfection efficiency (Fig. 3A), downregulation of MNK1, MCL1 and XIAP, as well as PARP cleavage (Fig. 3B), with no observed toxicity 24 h post transfection (Fig. 3A). Five hundred thousand control- and apMNKQ2-transfected cells were then injected in the tail vein of NOD.SCID mice (Fig. 3C), and 4 months post injection, mice were sacrificed and lung colonization was determined macro and microscopically. As shown, mice injected with apMNK2-transfected cells showed less lung tumor burden (Fig. 3D), which was significant at the level of lung weight (Fig. 3E). Histological examination of CK19- and H&E-stained sections confirmed less PDAC cell colonization in mice injected with apMNK2-transfected cells compared to control groups (Fig. 3F, G and Figure S3). Taken together, the sum of these findings presented herein underscore the potent anti-migratory effects of apMNKQ2 in PDX-derived PDAC cells.

### apMNKQ2 targets the cancer stem cell (CSC) compartment in PDAC

It is believed that the aggressiveness, metastatic capacity, and inherent chemoresistant nature of PDAC are multifactorial. However, there is growing evidence that these properties are mainly due to the existence of a subpopulation of highly tumorigenic cancer stem cells (CSCs) within the tumor [33]. In the case of PDAC, these cells represent the root of the tumor and, from a clinical perspective, only their elimination would guarantee tumor eradication and an improvement in overall survival. Currently, efforts are underway to develop therapies specifically targeting CSCs [11, 43, 50], with the goal of directly attacking these clinically relevant cells; however, to date, there are limited anti-CSC therapies that have reached the clinic or have shown promising effects in clinical trials.

**Fig. 4:**
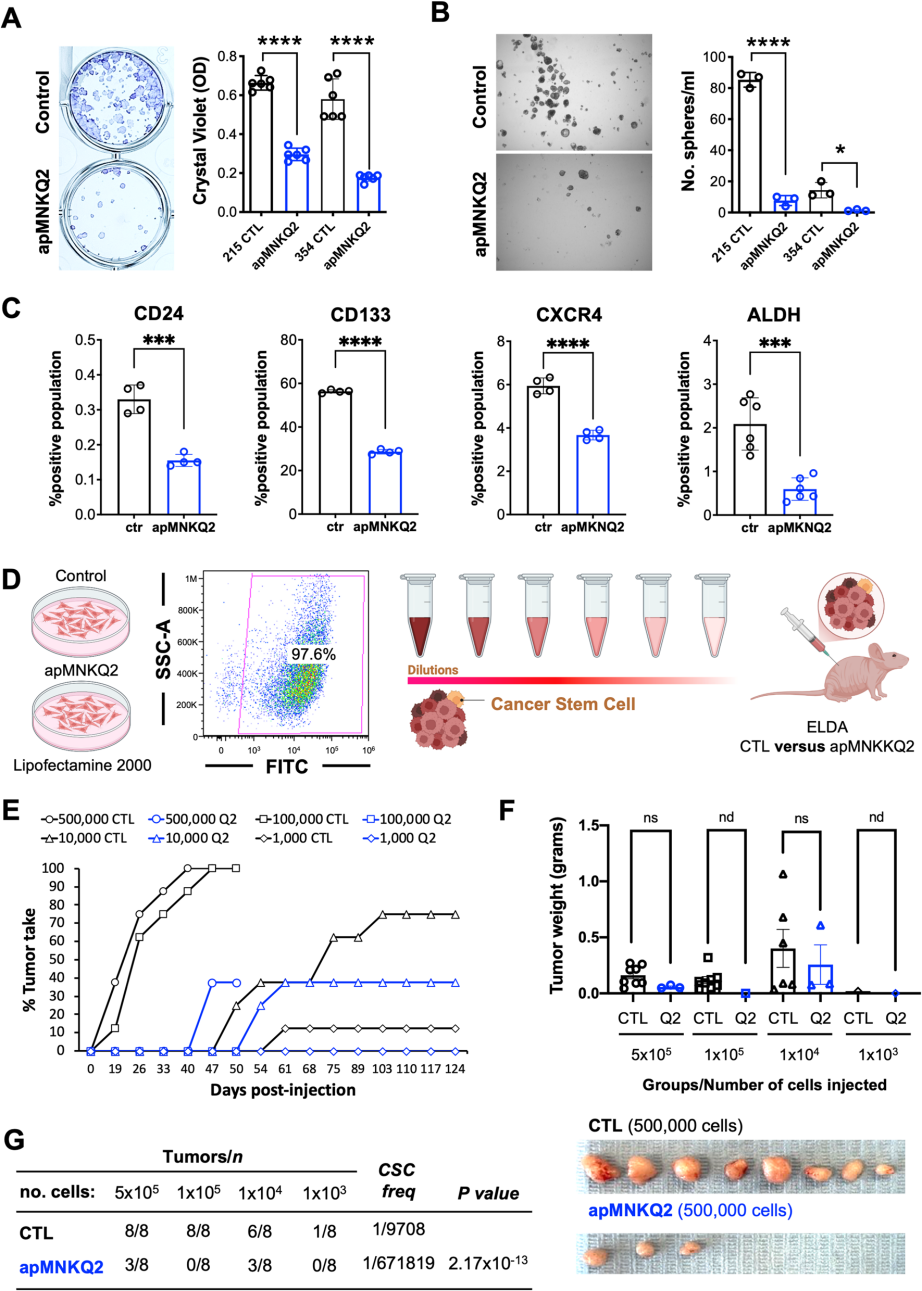
apMNKQ2 targets the CSC compartment in PDAC. **A, B** Left: Representative images of colonies (**A**) or spheres (**B**) in control- or apMNKQ2-transfected Panc354 cells. Right: Mean ± SEM in the crystal violet optical density (OD) (**A**) or number (no.) of spheres/ml (**B**) in control- or apMNKQ2-transfected Panc215 or Panc354 cells. * p < 0.05, **** p < 0.0001; as determined by one-sample t test. **C** Mean ± SEM of the percentage of CD24-, CD133-, CXCR4- or ALDH-positive cells in control- or apMNKQ2-transfected cells 24 h post transfection. *** p < 0.001, **** p < 0.0001; as determined by one-sample t test. **D** Schematic of the transfection groups, apMNKQ2-FITC transfection efficiency determined by flow cytometry and subsequent dilution of cells for ELDA determination. **E** Percentage of tumor take (number of tumors confirmed/number of injections) for subcutaneously injected control (CTL)- and apMNKQ2 (Q2)-transfected cells was determined over the course of 124 days. **F** Tumor weight (g) o tumors extracted at indicated times in (**E**). ns = not significant; nd = not determined. **G** CSC frequencies determined using the extreme limiting dilution analysis algorithm (http://bioinf.wehi.edu.au/software/elda/index.html) (left, 95% CI) and images of resected tumors for the dilution 5 × 10^5^ (right)

Given the potent anti-proliferative and anti-migratory properties of apMNKQ2, we asked whether apMNKQ2 specifically targets the CSC subpopulation within PDX-derived PDAC cell lines. Indeed, the role of MNK1 in controlling CSCs characteristics has been highlighted across various malignancies, including breast cancer, chronic myeloid leukemia, and glioblastoma, where its expression influences stemness [4, 29, 39, 48]; however, no study has yet to demonstrate a similar correlation in pancreatic CSCs. Towards this end, RNAseq analysis of Panc354 and Panc215 apMNKQ2-treated cells versus control cells was performed and GSEA revealed a reduction in the normalized enrichment scores in multiple stem cell signatures for apMNKQ2-treated cells (Figure S4A-B), indicating that apMNKQ2 negatively affects stem-associated pathways. In line with these results, sphere- and colony-formation assays revealed a significant reduction in CSC clonogenic capacity and self-renewal upon apMNKQ2 transfection of Panc215 and Panc354 cells (Fig. 4A, B). Concomitantly, flow cytometry analysis demonstrated a decrease in the CSC cell-surface markers CD24, CD133 and CXCR4, the latter of which has been linked to an invasive CSC population [32], as well as aldehyde dehydrogenase activity (ALDH^high^), measured using the Aldefluor assay (Fig. 4C).

To functionally validate the impact of apMNKQ2 on CSCs, we performed an in vivo extreme limiting dilution assay (ELDA), the gold standard to determine the anti-CSC potential of a compound. To this end, Panc354 cells were transfected with 100 nM apMNKQ2 for 24 h, transfection efficiency was confirmed, cells were diluted and 5 × 10^5^, 1 × 10^5^, 1 × 10^4^ or 1 × 10^3^ cells were injected subcutaneously in athymic nude mice (Fig. 4D). Over the course of 4 months, the percentage of tumors that formed (i.e., tumor take) as a function of the number of injections performed (*n* = 8/dilution) were determined. For each dilution, mice were sacrificed when 100% tumor take was achieved in one of the two groups and/or when a humane endpoint sacrifice for one of the two groups was required. For all dilutions, apMNKQ2-transfected cells showed delayed tumor appearance with significantly fewer tumors compared to control-transfected cells (Fig. 4E). Importantly, tumors that did appear from apMNKQ2-transfected cells were not significantly smaller (Fig. 4F), indicating that the effects observed were not due to decreased proliferation, but rather an inhibition in the tumor initiating capacity of the cells. This translated to a 692-fold decrease in CSCs frequency following treatment with apMNKQ2, as calculated using the Extreme Limiting Dilution Analysis (https://bioinf.wehi.edu.au/software/elda/) (Fig. 4G). Together, these findings indicate a central role for MNK1 in the maintenance of PDAC CSCs in vivo.

### apMNKQ2 inhibits PDAC tumor growth in an orthotopic model

**Fig. 5:**
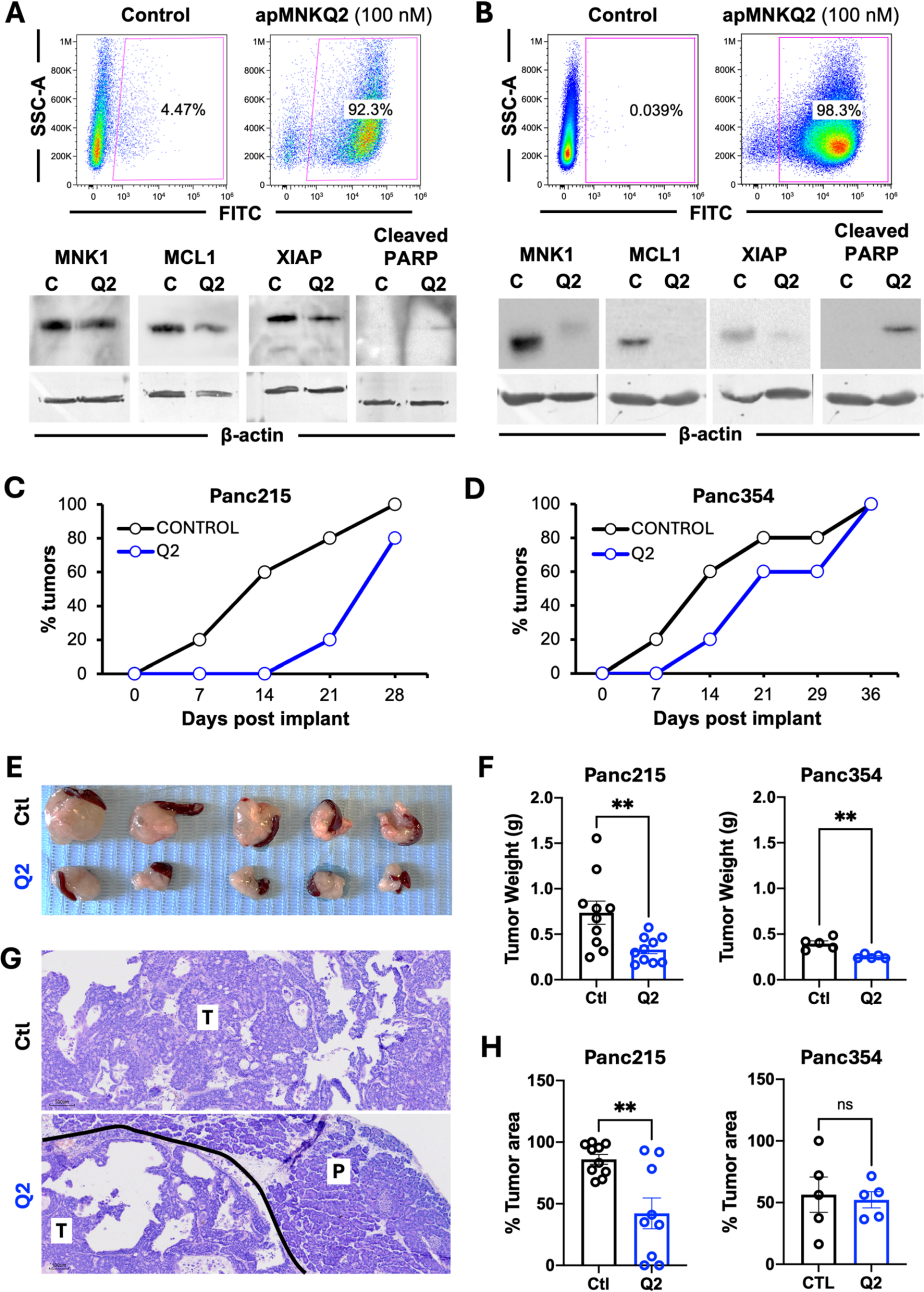
apMNKQ2 inhibits PDAC cell orthotopic tumor growth. **A, B** Representative dot plots of the percentage of FITC-positive (top) Panc215 (**A**) or Pan354 (**B**) cells 24 h post transfection with control or apMNKQ2-FITC (100 nM). WB analysis of MNK1, MCL1, XIAP or cleaved PARP protein levels in control- (C) or apMNKQ2-FITC (Q2)-transfected Panc215 (**A**) or Pan354 (**B**) cells (bottom). β-actin is included as a loading control. Percentage of tumor take (number of tumors confirmed by palpation/number of injections) for orthotopically-injected control- or apMNKQ2 (Q2)-transfected Panc215 (n = 10) (**C**) or Pan354 (n = 5) (**D**) cells was determined over the course of 28 or 36 days, respectively. **E** Representative images of Panc215 orthotopic tumors extracted at day 28 post injection. **F** Tumor mean weight (g) ± SEM of orthotopically-injected cells from control (Ctl)- or apMNKQ2 (Q2)-transfected Panc215 (n = 10) or Pan354 (n = 5) cells. **G** Representative micrographs of H&E-stained tumors from E. T = tumor; P = normal pancreas. **H** Percentage of tumor area in pancreata from control (Ctl)- or apMNKQ2 (Q2)-transfected Panc215 (n = 10) or Pan354 (n = 5) cells, calculated as the area of tumor tissue divide by the total area of the pancreas

Based on the in vitro and ELDA results, the antitumorigenic potential of apMNKQ2 was further evaluated in vivo by transfecting Panc354 and Panc215 cells, and orthotopically injecting them in NOD.SCID mice to evaluate their tumor initiating capacity in the pancreas. Transfection efficiency (Fig. 5A, B) (upper panels), downregulation of MNK1, MCL1 and XIAP, and PARP cleavage were determined (Fig. 5A, B) (lower panels), confirming efficient transfection in both PDX-derived cell lines. Over the course of 28–36 days, cells transfected with apMNKQ2 generated tumors more slowly than those generated by control-transfected cells, as determined by palpation, with a significant delay in tumor onset for Panc215 cells (Fig. 5C, D). Upon sacrifice at 28- or 36-days post implantation, a significant reduction in tumor size and weight was observed (Fig. 5E-F). Histological analysis of tumors was performed to determine the total tumor area as a percentage of the total area of the pancreas (Fig. 5G, H). Only for apMNKQ2-transfected Panc215 cells did we observe a significant reduction in total tumor area (Fig. 5H), which is in line with the increased delay in tumor take post implantation and the greater difference between tumor size and weight at the time of sacrifice between the control and apMNKQ2-transfected groups. Of note, Panc215 is a more proliferative PDX-derived cell line, able to colonize approximately 100% of the pancreas in 28 days. Thus, the difference between the control and apMNKQ2-transfected Panc215 cells was more evident than with Panc354, which is a less proliferative, but highly metastatic, tumor cell line. Nonetheless, these results indicate that the efficient transfection of PDAC cells with apMNKQ2 reduces their capacity to efficiently proliferate in the pancreas of mice.

To determine the effect on metastasis, livers from mice were stained for CK19 and the human-specific nuclear probe Nucleoli NM95 to evaluate the presence of disseminated tumor cells (Figure S5). Consistent with prior reports by Hidalgo and colleagues describing inefficient metastatic dissemination with PDAC orthotopic PDX models [17], no human tumor cells were detected in the liver in either control or apMNKQ2 groups, supporting the known limitation of these models in recapitulating metastatic spread.

### Free apMNKQ2 efficiency targets PDAC CSCs

**Fig. 6:**
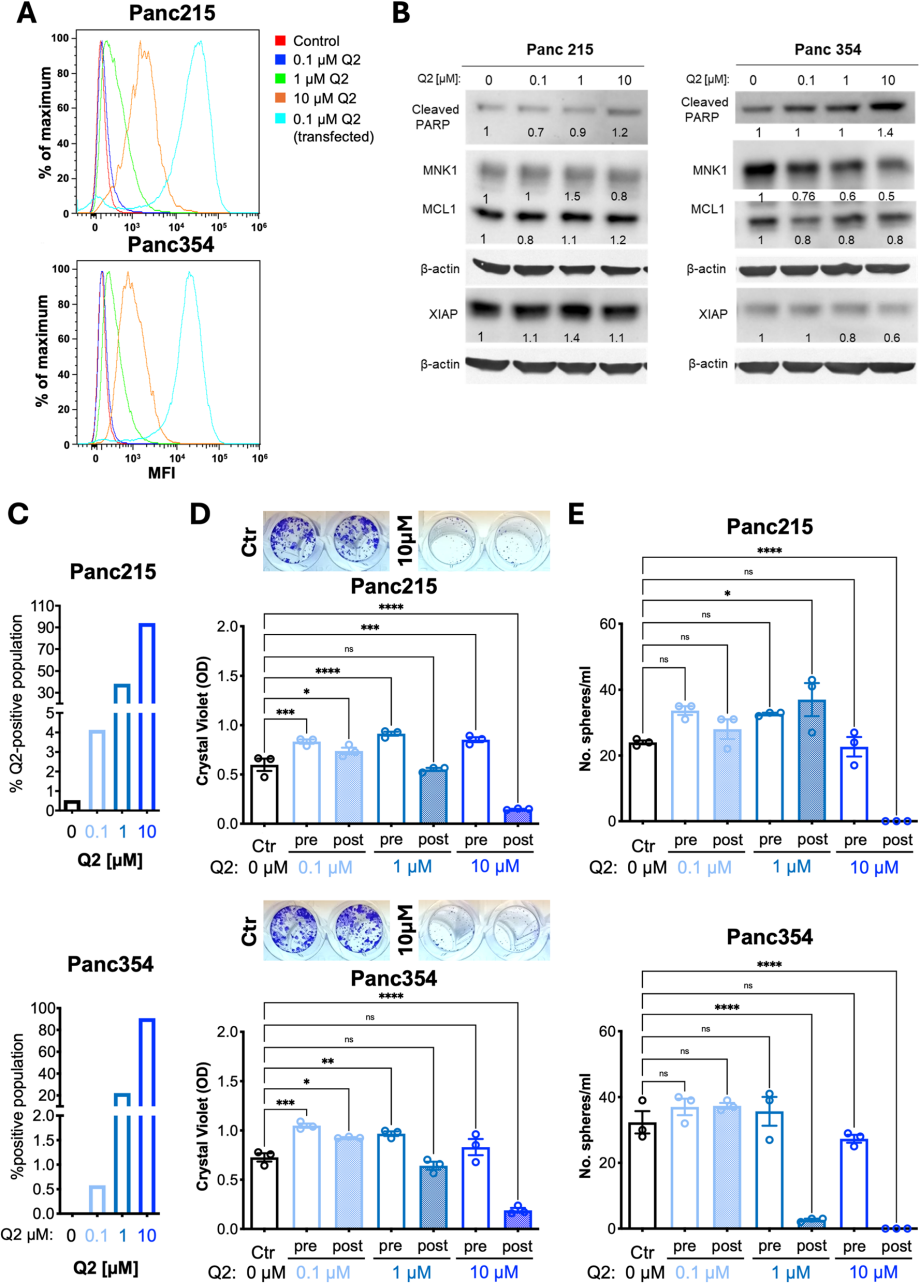
Free apMNKQ2 efficiently targets PDAC CSCs. **A** Mean fluorescence intensity (MFI) in control or apMNKQ2-FITC (Q2)-treated (0.1, 1 or 10 µM) Panc215 (top) or Pan354 (bottom) cells compared to cells transfected with 0.1 µM apMNKQ2-FITC (Q2). **B** WB analysis of cleaved PARP, MNK1, MCL1 or XIAP protein levels in control-(0 µM) or apMNKQ2- (0.1, 1 or 10 µM) treated Panc215 or Panc354 cells. β-actin is included as a loading control. Fold change in the levels of β-actin-normalized protein expression levels are shown below each blot. **C** Percentage of FITC-positive cells in control or apMNKQ2-FITC (Q2)-treated (0.1, 1 or 10 µM) Panc215 (top) or Pan354 (bottom) cells 24 h post treatment. **D, E** Panc215 (top) or Panc354 cells (bottom) were either pre-treated with increasing doses of apMNKQ2 (0.1, 1 or 10 µM) for 24 h prior to establishing colonies or spheres (i.e., pre), or treated when colonies or spheres were established (i.e., post). **D** Top: Representative images of colonies 7 days post control (Ctr)- or apMNKQ2-treatment (10 µM). Bottom: Mean ± SEM of crystal violet optical density (OD) of colonies 7 days control (Ctr)- or apMNKQ2 pre or post treatment (0.1, 1 or 10 µM). **E** Mean number (no.) of spheres/ml ± SEM in control (Ctr)- or pre or post apMNKQ2-treated (0.1, 1 or 10 µM) cells 5 days post sphere initiation. * p < 0.05, ** p < 0.01, *** p < 0.001, **** p < 0.0001, ns = not-significant; by One-way ANOVA with Dunnett's multiple comparison test

While cationic-lipid transfection reagents are efficient for delivering nucleic acids to cells in vitro, in vivo transfection of nucleic acids remains a significant challenge. With the main and final goal of this study being the treatment of established tumors with apMNKQ2 in vivo, we tested whether non-lipofectamine conjugated apMNKQ2 could efficiently enter into PDAC cells and have the same biological consequences. To this end, Panc215 and Panc354 cells were treated with free apMNKQ2 at 0.1, 1.0 and 10 µM for 24 h and compared to cells transfected with 100 nM of apMNKQ2 using Lipofectamine. A clear dose dependent increase in the amount of apMNKQ2, quantified via qPCR (Figure S6A), and in the mean intensity of FITC was noted; however, 10 µM of apMNKQ2 did not achieve the same levels of internalization (as measured by mean intensity) as with lipofectamine, as would be expected (Fig. 6A). Nonetheless, the downstream effects on MNK1, MCL1, XIAP and cleaved PARP with 10 µM of free apMNKQ2 were similar to what we had observed with transfection (Fig. 6B), and the percentage of cells positive for the FITC-labeled aptamer increased in a dose dependent manner, with approximately 100% of the cells positive with 10 µM (Fig. 6C). Of note, at 10 µM apMNKQ2, we observed a preference for accumulation of the aptamer in CD133-positive and CXCR4-positive cells (Figure S6B).

To evaluate the functional effects of free apMNKQ2, cells were either pre-treated with increasing doses of apMNKQ2 for 24 h prior to establishing colonies or spheres (i.e., pre), or treated when colonies or spheres were established (i.e., post). For both assays, only 10 µM apMNKQ2 added when colonies or spheres were established significantly reduced the clonogenic capacity (Fig. 6D) and self-renewal capacity (Fig. 6E) of Panc215 and Panc354 cells. At the level of sphere formation, the effect was maintained in time as observed in second generation sphere formation assays (Figure S6C). Thus, free apMNKQ2 is also able to efficiently enter into PDAC cells and target the CSC compartment, and although cationic-lipid transfection increases the total amount of apMNKQ2 entering cells, the fraction of cells that actually take up the aptamer is more critical than the overall quantity delivered.

### Biodistribution, toxicity and pharmacokinetics (PK) of free apMNKQ2 in vivo

Previously, we demonstrated that apMNKQ2 can be administered in vivo without the jetPEI^®^ transfectant [9]. To confirm this observation and carry out in vivo trials in pancreatic cancer models, the optimal route of administration of apMNKQ2, either alone or conjugated with the jetPEI^®^ transfectant, was first established. For this, we monitored in real time the fluorescence emitted by a FITC-labeled aptamer using an In vivo Imaging System (IVIS) after administering a single dose of 1.7 mg/Kg of apMNKQ2-FITC retro-orbitally (r.o.) or intraperitoneally (i.p.) to CD1 Swiss mice. The results showed that i.p. administration led to faster distribution kinetics, but after 4 h, both routes achieved systemic distribution (Figure S7). Although apMNKQ2-FITC conjugated with jetPEI^®^ exhibited higher fluorescent signal intensity throughout the experiment, the unconjugated apMNKQ2-FITC also demonstrated homogeneous and efficient distribution. It is important to note that rodent well-being indicators (weight, temperature, motor activity, etc.) were monitored and no adverse effects were observed during the course of the experiment nor up to 5 days post injection.

**Fig. 7:**
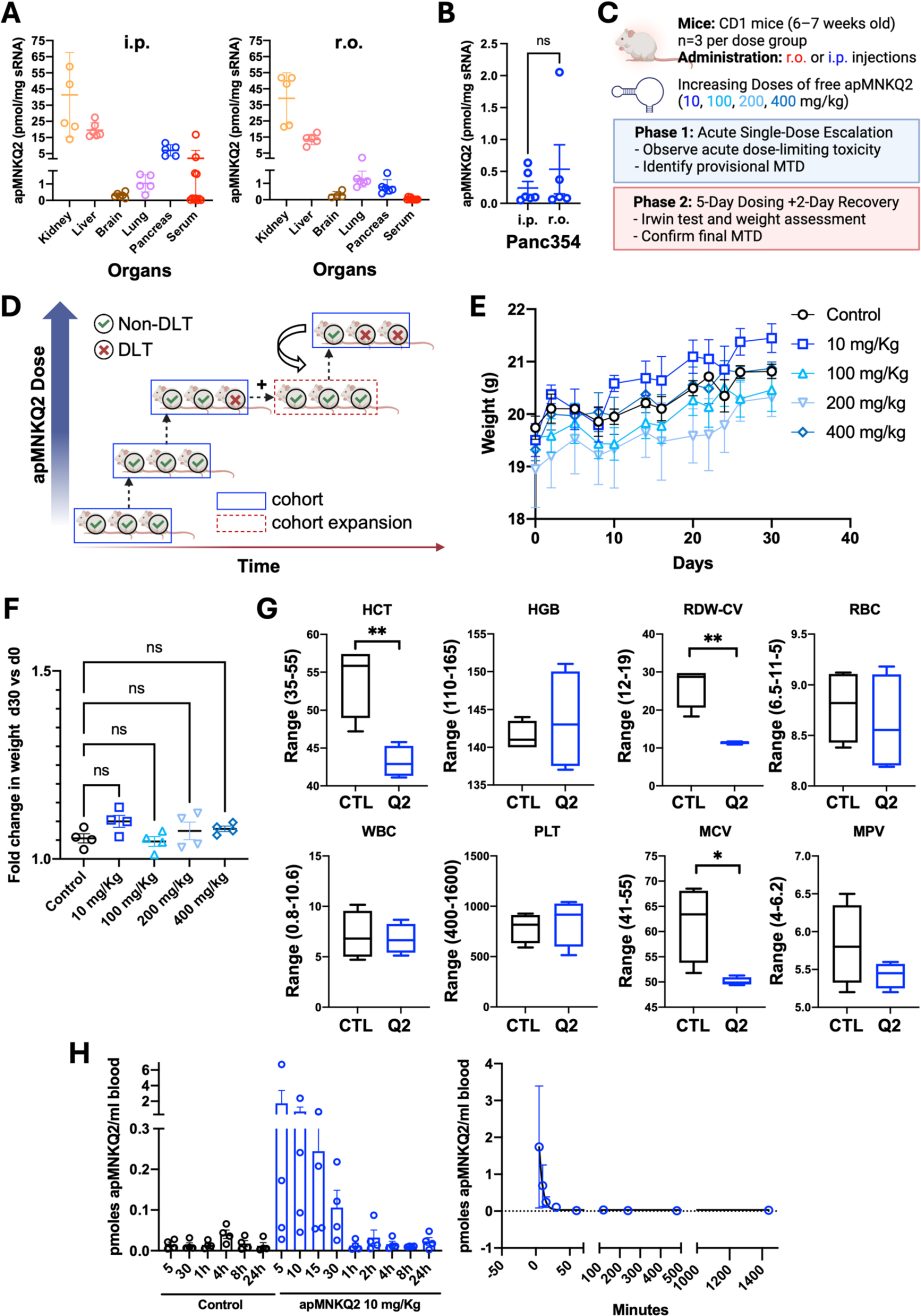
Biodistribution, toxicity and pharmacokinetics of free apMNKQ2 in vivo. **A** Mean pmol/mg of RNA of apMNKQ2 ± SEM, determined by qPCR, in kidney, liver, brain, lung, pancreas and serum 4 h post i.p. or r.o. administration of 10 mg/Kg of free apMNKQ2. (n = 5 mice per group). **B** Mean pmol/mg of RNA of apMNKQ2 ± SEM, determined by qPCR, in Panc354 tumors 4 h post i.p. or r.o. administration of 10 mg/Kg of free apMNKQ2. **C** Schematic for the determination of the maximum tolerated dose (MTD) in CD1 mice following an acute (Phase 1) and 5-day (Phase 2) dosing strategy. **D** Illustration of the modified ‘‘3 + 3’’ study design. Each box represents a cohort comprising the indicated number of mice treated at a given dose level. DLT = dose-limiting toxicity. **E** Mean weight (g) ± SEM of mice over the course of 30 days of continuous i.p. treatment with 0 (control), 10, 200, 200 or 400 mg/Kg of free apMNKQ2. (n = 5 mice per group). **F** Mean fold change in weight ± SEM of mice at the end of the dosage time-course experiment shown in (**E**), comparing d30 with d0, ns = not-significant; determined by One-way ANOVA with Dunnett's multiple comparison test. **G** Analysis of the indicated hematocrit parameters in mice treated with 0 (CTL) or 400 mg/Kg of free apMNKQ2 (Q2) for 30 consecutive days. Box plots indicate the mean ± SEM of the levels determined. (n = 5 mice per group). Established normal range parameters are indicated. *HCT* hematocrit, *HGB* hemoglobin, *RDW-CV* red cell distribution width-coefficient of variation, *RBC* reb blood cell count, *WBC* white blood cell count, *PLT* platelet count, *MCV* mean corpuscular volume, *MPV * mean platelet volume. *p < 0.05; **p < 0.01 relative to control; as determined by one-sample t test. **H** Left: Mean pmol/ml of blood of apMNKQ2 ± SEM, determined by qPCR, in serum of mice treated with 0 (CTL) or 10 mg/Kg of free apMNKQ2 and extracted at the indicate times. (n = 4 mice per group). Right: Kinetics graph of the pharmacokinetics of 10 mg/Kg of free apMNKQ2 as a function of time and graphed as the mean pmol/ml of blood of apMNKQ2 ± SEM

Next, the distribution of apMNKQ2 following i.p. or r.o. administration of 10 mg/Kg of free apMNKQ2 was evaluated by quantifying the aptamer via qPCR at 4 h post-administration in serum, kidney, liver, brain, lung and pancreas (Fig. 7A). An oral gavage group was also included; however, no aptamer was detected in any organ or in the serum of animals that received oral administration, indicating poor gastrointestinal absorption (data not shown). Comparing i.p. vs. r.o. administration, in both cases the aptamer accumulated in the liver and kidneys, consistent with clearance through the bloodstream. Among the other organs examined, apMNKQ2 accumulation in the pancreas was detected only in the i.p. group. Interestingly, apMNKQ2 was undetectable or barely detectable in serum samples at 4 h post r.o. or i.p. injection, indicative of a brief circulating half-life (Fig. 7A). Lastly, apMNKQ2 was detected in subcutaneously-implanted Panc354 PDX tumors, with no significant differences observed between i.p. vs. r.o. administration (Fig. 7B).

Determining the maximum tolerated dose (MTD) in mouse models is crucial for gauging the highest safe dose of a therapeutic agent before it causes unacceptable side effects. Moreover, knowing the MTD helps optimize dosing regimens and improves the reliability of translating findings to human clinical trials. The MTD was determined with increasing doses of free apMNKQ2: 10, 25, 50, 100, 200 and 400 mg/Kg in CD1 mice (6–7 weeks old, n = 3 per group) injected r.o. or i.p. (Phase 1) and its prolonged toxicity was followed over a 5-day dosing with a 2-day recovery period (Phase 2) (Fig. 7C). The Phase 1 study showed that none of the doses tested, regardless of the administration route, proved toxic, using our previously published modified “3 + 3” study design [24] (Fig. 7D).

For the Phase 2 long-term study, i.p. administration was used as this route of administration showed specific advantages over r.o. administration in the aforementioned studies, and most importantly, was more amenable to daily treatments. The Phase 2 study showed that 5-day consecutive i.p. administration of apMNKQ2 over 30 days was well tolerated by 6- to 8-week-old female CD-1 mice across all doses tested as determined by the Irwin Test, typically used to assess a compound’s influence on both behavior and physiological processes (behaviorally, neurologically and autonomically). The Irwin Test helps to identify possible toxicity and guides the selection of doses for targeted therapeutic purposes [40]. A comprehensive assessment of overall health, survival, physical appearance (e.g., grooming, hair quality and shine), excitation, stereotypy, motor activity, sedation, pain, autonomy, respiration and hypo-/hyperthermic parameters was carried out in CD-1 mice, with an initial weight of 20 g (± 1.5 g). The mice were weighed 2–3 times weekly to monitor weight gain or loss during the 30-day treatment period (Fig. 7E). No mouse showed signs of toxicity based on the Irwin test, and none exhibited a weight loss greater than 30% of its original body weight during the treatment. At the conclusion of the experiment, an average increase in weight at d30 versus d0 was observed for all mice, with no significant differences when compared to control-treated mice (Fig. 7E, F). Therefore, the MTD was determined to be = 400 mg/Kg, which is also the maximum feasible dose (MFD) based on synthesis constraints.

Once the administration route and MTD had been established, a pathological analysis of organs and hematological analyses were performed. Following necropsy, all organs appeared macroscopically normal, with no differences observed between treated and untreated mice. From each mouse, (1) brain, intestine, heart and lungs, and (2) liver, spleen, kidney and pancreas were collected, placed in two cassettes, and fixed overnight in 4% PFA, embedded in paraffin, sectioned and stained with hematoxylin and eosin (H&E) for pathological analysis. The histology of each organ from treated mice was compared with that of control mice in a general assessment. After examining every organ in each group, no signs of inflammation, necrosis, fibrosis, degeneration or hyperplasia were found in any of the samples. A comprehensive hematological assessment was also conducted using the Element HT5 veterinary hematology analyzer. Whole blood was isolated from mice treated with 400 mg/Kg apMNKQ2 for 4 weeks, and the parameters shown in Fig. 7G and Figure S8 were analyzed. Although some significant differences were observed between control mice and apMNKQ2-treated mice, all hematological parameters fell within normal ranges for mice, indicating that apMNKQ2 does not induce adverse hematological effects.

Lastly, to determine the pharmacokinetics (PK) of apMNKQ2 in blood, 10 mg/Kg of apMNKQ2 was administered i.p. to establish the aptamer’s PK profile. Blood samples were collected from both control and apMNKQ2-treated mice at 5, 10, 15 and 30 min up to 24 h, followed by qPCR quantification of apMNKQ2. As shown in Fig. 7H, apMNKQ2 had a very short half-life in blood, reaching its peak at 5–10 min post injection. One hour after administration, apMNKQ2 levels were the same as in control mice, indicating a very brief circulating half-life, in line with our results comparing i.p. versus r.o. administration (Fig. 7A).

### Efficacy of free apMNKQ2 in PDX models in vivo

**Fig. 8:**
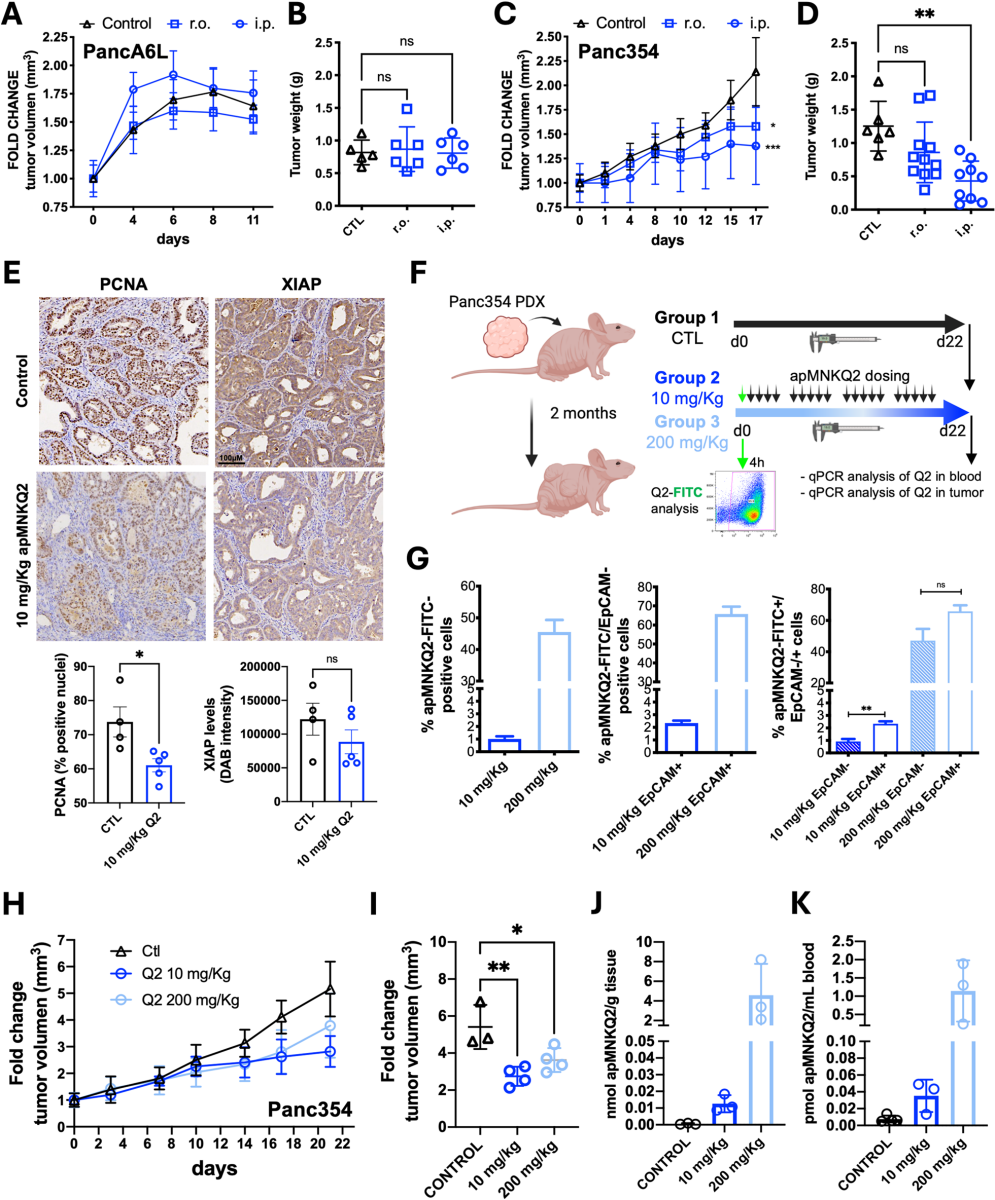
Free apMNKQ2 biodistribution and efficacy in vivo. **A** Mean fold change in tumor volume ± SEM in mice bearing PancA6L PDXs and treated with 10 mg/Kg of apMNKQ2 retro orbitally (r.o.) or intraperitoneally (i.p.) over the course of 11 days and compared to d0 (n = 5–6 tumors/group). **B** Tumor weight (g) ± SEM from (A) determined on day 11 post treatment initiation. ns = not significant; as determined by one-way ANOVA with Dunnett post-test, compared to control (Ctl). **C** Mean fold change in tumor volume ± SEM in mice bearing Panc354 PDXs and treated with 10 mg/Kg of apMNKQ2 retro orbitally (r.o.) or intraperitoneally (i.p.) over the course of 17 days and compared to d0 (n = 6–11 tumors/group). *p < 0.05, ***p < 0.001; as determined by one-way ANOVA with Dunnett post-test, compared to control (Ctl). **D** Tumor weight (g) ± SEM from (**C**) determined on day 17 post treatment initiation. **p < 0.01, ns = not significant; as determined by one-way ANOVA with Dunnett post-test, compared to control (Ctl). **E** Top: Representative immunohistochemical images of PCNA and XIAP staining in Panc354 tumors from (C). Bottom: Mean percent positive PCNA nuclei ± SEM or mean XIAP levels ± SEM determined for extracted tumor. *p < 0.05, ns = not significant; as determined by one-way ANOVA with Dunnett post-test, compared to control (Ctl). **F** Experimental set-up for in vivo experiments using subcutaneously implanted Panc354 PDXs and treatment with control (Group 1), free apMNKQ2-FITC (d0) or apMNKQ2 (10 mg/Kg i.p.; daily, Group 2) or free apMNKQ2-FITC (d0) or apMNKQ2 (200 mg/Kg i.p.; daily, Group 3) for 22 days. **G** Analysis of apMNKQ2-FITC 4 h post injection in all cells extracted from the tumor, in EpCAM-positive cells, or in EpCAM-negative versus EpCAM-positive cells, as determined by flow cytometric analysis. **p < 0.01, ns = not significant; as determined by one-way ANOVA with Dunnett post-test, compared to control (Ctl). **H** Mean fold change in tumor volume ± SEM in mice bearing Panc354 PDXs and treated with 10 or 200 mg/Kg of apMNKQ2 intraperitoneally (i.p.) over the course of 22 days and compared to d0 (n = 3–4 tumors/group). **I** Fold change in tumor volume (mm^3^) ± SEM from (**H**) determined on day 22 post treatment initiation. *p < 0.05, **p < 0.01; as determined by one-way ANOVA with Dunnett post-test, compared to control (Ctl). **J-K** Mean nmol/g or pmol/ml of apMNKQ2 ± SEM, determined by qPCR, in the tumors (**J**) or serum (**K**) on day 22, respectively (4 h post final i.p. administration of 10 or 200 mg/Kg of free apMNKQ2, n = 3 samples per group)

The in vivo efficacy of free apMNKQ2 was initially assessed in established PDAC tumors (~ 150 mm^3^) at a dose of 10 mg/kg, administered 5 days per week via i.p. or r.o. injection. Two PDX models were tested: Panc354, which displayed the highest sensitivity to apMNKQ2, and PancA6L, which was highly resistant. In line with our IC_50_ studies (Fig. 1D), in PancA6L tumors, no reduction in tumor volume or tumor weight was observed with either route of administration (Fig. 8A, B). In contrast, Panc354 tumors exhibited a marked reduction in growth beginning after 1.5 weeks of treatment, particularly with i.p. dosing (Fig. 8C). At the endpoint, both treatment groups (i.p. and r.o.) showed a significant decrease in tumor volume compared to controls, with no significant differences between the two routes of administration (Fig. 8C). However, a significant reduction in tumor weight was observed only with i.p. administration (Fig. 8D). Immunohistochemical (IHC) analysis of apMNKQ2 i.p.-treated tumors revealed a modest but significant decrease in PCNA staining (indicating reduced cell proliferation) and a non-significant but appreciable decrease in XIAP expression (Fig. 8E).

Because higher doses of apMNKQ2 had shown no toxicity in prior experiments (Fig. 7), we next evaluated a 20-fold higher dose (200 mg/Kg) in the Panc354 PDX model. Again, once tumors reached ~ 150 mm^3^ in volume, apMNKQ2 was administered i.p. at 10 or 200 mg/Kg (Fig. 8F). To assess tumor uptake differences between the two doses, the first dose was given with apMNKQ2-FITC to monitor intra tumoral aptamer distribution by flow cytometry. Approximately 60% of bulk tumor cells (Fig. 8G**, **left) and 65% of EpCAM-positive human tumor cells (Fig. 8G**, **middle) were FITC-positive following administration of 200 mg/kg apMNKQ2-FITC, an ~ 28-fold increase over tumors treated with 10 mg/kg, demonstrating that higher doses improve aptamer delivery to the tumor, and specifically to the human tumor cell compartment, although the aptamer also enters into EpCAM-negative cells (i.e., fibroblasts, immune cells and endothelial cells) **(**Fig. 8G**, **right). Surprisingly, however, treatment with 200 mg/Kg did not improve the antitumor efficacy of apMNKQ2. No significantly enhanced reduction in tumor volume was observed in mice treated with 200 mg/Kg compared to the 10 mg/Kg dose over a 21-day period (Fig. 8H, I). At sacrifice (4 h after the final injection), qPCR analysis confirmed higher levels of apMNKQ2 in both tumor tissue and blood in the 200 mg/Kg group relative to the 10 mg/Kg group (Fig. 8J, K). Despite this increased accumulation, 200 mg/Kg of apMNKQ2 was no more effective, suggesting that 10 mg/kg is the maximum efficacious dose, although further studies are still needed to confirm this result and determine the minimum efficacious dose. If confirmed, however, this lower dose could hold translational advantages for manufacturing and dosing considerations in humans.

## Discussion

In this study, we aimed to assess the therapeutic value of apMNKQ2 against PDAC. Our results consistently showed that MNK1 is expressed in all the PDX-derived PDAC cell lines tested, with varying levels of expression, and that apMNKQ2 markedly decreases its protein levels. This downregulation translated into robust antitumor effects in vitro, as demonstrated by reduced cell viability and proliferation, modulation of apoptosis-associated proteins, and inhibition of cell cycle factors. Regarding the latter, our findings are consistent with previous studies that have demonstrated the importance of MNK1 in cell cycle regulation and protein translation in cancer cells. MNK1 is required for the expression of critical cell cycle regulators such as E2F1, FoxM1 and Wee1 in soft tissue sarcoma cancer progression [40], E2F1 and FoxM1 are transcription factors that play pivotal roles in cell cycle checkpoints, preventing cells with DNA damage from entering mitosis. The downregulation of these proteins upon MNK1 inhibition suggests a mechanism by which apMNKQ2 may exert its antitumor effects, potentially leading to cell cycle arrest and reduced tumor cell proliferation. These findings align with our observations of decreased CDK1 and Cyclin B levels following apMNKQ2 treatment, further supporting the hypothesis that MNK1 inhibition disrupts critical cell cycle regulators, thereby impeding cancer cell progression. Importantly, these effects were seen across multiple primary PDX-derived PDAC cell lines, reinforcing the notion that MNK1 functions as a plausible and perhaps undervalued node of oncogenic signaling in PDAC.

A notable finding was the capacity of apMNKQ2 to reduce PDAC cells’ migratory properties. GSEA showed that high MKNK1 expression in patients and PDX-derived cell lines correlates with enhanced EMT and angiogenesis pathways, which are essential for metastasis. Indeed, our in vitro assays revealed that apMNKQ2 inhibited PDAC cell in vitro cell migration and impacted MMP9 secretion, together with diminished vascular tubule formation by endothelial cells when exposed to conditioned media from apMNKQ2-treated cells. Notably, these results were validated in vivo, where apMNKQ2-transfected cells were less capable of colonizing the lungs of injected mice, relative to controls. Collectively, these data underscore the importance of targeting MNK1 to inhibit PDAC cell dissemination and colonization, a necessary step during metastasis, the primary determinant of PDAC-related mortality. Further studies using more biologically relevant genetically-engineered mouse models of PDAC, where true metastatic dissemination can be evaluated, are still required to fully elucidate the anti-metastatic capacity of apMNKQ2.

Another important finding was the demonstration that apMNKQ2 targets the PDAC CSC subpopulation. Mounting evidence supports the concept that CSCs, which typically make-up only a small fraction of tumor cells, are the principal drivers of tumorigenesis, metastasis, therapeutic resistance and relapse. Here, apMNKQ2 reduced the expression of multiple stem-related pathways and the clonogenic and self-renewal capacities of PDAC cells, diminished the expression of canonical CSC surface markers (including ALDH and the metastatic CSC marker CXCR4), and most importantly, substantially decreased the tumor-initiating frequency in vivo. The effect on ALDH is of particular interest as this is consistent with previous work by M. K. Evans et al*.* [19], demonstrating that MNK1 regulates XIAP expression and downstream NF-κB signaling, thereby promoting ALDH-positive CSCs in breast cancer. Thus, inhibition of MNK1 by apMNKQ2 would be expected to affect ALDH activity. Finally, the gold-standard ELDA confirmed that CSCs were significantly depleted after apMNKQ2 treatment, highlighting MNK1’s pivotal role in targeting this stem-like and aggressive tumor cell subpopulations. This is in line with the higher expression of *MKNK1* in PDAC patients with a basal subtype.

To evaluate the amenability of apMNKQ2 for clinical usage, we explored the potential of administering apMNKQ2 without transfection reagents. Although cationic lipids facilitated higher aptamer uptake in vitro, free apMNKQ2 at higher concentrations (i.e., 10 µM) was also taken up efficiently by PDAC cells, achieving comparable modulation of MNK1 and its downstream targets, compared to transfected apMNKQ2. We reasoned that the fraction of cells taking up the aptamer, rather than the total amount taken up, is key for its antitumor efficacy. This is particularly true in light of the observation that the CSC CD133 +/CXCR4 + population had a higher propensity (~ 1.8-fold) for uptake of free apMNKQ2, which likely was the reason for the potent effects observed in the clonogenic and sphere-forming capacity of PDAC cells treated with free apMNKQ2. Further studies are necessary, however, to determine the mechanism by which apMNKQ2 preferentially accumulates within CSCs.

Our biodistribution and pharmacokinetics studies also confirmed that free apMNKQ2 can reach the systemic circulation, accumulates in major organs and is detectable within PDAC tumors in mice. Notably, i.p. administration facilitated the aptamer’s accumulation in the pancreas, further supporting the translational feasibility of this route. Although it exhibits a short circulation time, repeated intraperitoneal dosing was well-tolerated up to 400 mg/kg, revealing a wide therapeutic window and no major histopathological abnormalities. Thus, free apMNKQ2 has low to no toxic effects in vivo, within the parameters measured in this study.

Finally, in PDX tumor models, free apMNKQ2 significantly delayed tumor growth in mice xenografted with the sensitive PDAC PDX Panc354, while treatment of the resistant PDX PancA6L yielded no substantial benefit. This is an interesting observation and suggests that the overall levels of MNK in the tumor biopsies may need to be taken into consideration to best identify those patients that will benefit from future apMNKQ2-based treatment approaches. Dose escalation from 10 mg/kg to 200 mg/kg improved intratumoral aptamer delivery but did not enhance the antitumor effect, implying that 10 mg/kg may already be at or near the maximal biologically effective dose for this context. This observation holds promise for eventual clinical translation, as lower doses can simplify manufacturing and administration without compromising efficacy. Although additional studies will be needed to pinpoint the optimal dosing regimen, these findings pave the way for the development of aptamer-based, MNK1-targeting therapies against PDAC. Likewise, we do not rule out possible future modifications, such as aptamer pegylation or nanoparticle encapsulation, which might prolong the half-life of apMNKQ2. The latter could reduce administration times, which in this study were 5 times per week. Likewise, a prolonged half-life of apMNKQ2 in circulation could also facilitate its enhanced entry into CSCs in vivo, an aspect that was not evaluated in the tumors treated with free apMNKQ2.

## Conclusions

Taken together, our data provide extensive preclinical validation that targeting MNK1 through apMNKQ2 not only suppresses key PDAC in vitro properties, but also exerts profound anti-migratory/colonization and anti-CSC effects in vivo. By concurrently impacting multiple pathways responsible for survival, proliferation, stemness and invasion, apMNKQ2 presents an interesting candidate for further development as a novel therapeutic approach in PDAC. The ability of apMNKQ2 to specifically affect CSCs is of particular relevance, as standard treatments that fail to eliminate this subpopulation often yield tumor regrowth and metastatic spread. Future work will focus on optimizing the dose and schedule, exploring combination regimens with existing standards of care, and potentially investigating aptamer conjugation approaches to improve delivery and therapeutic index. Moreover, patients with high levels of MNK1 may be more susceptible to anti-MNK1 inhibition. Ultimately, this study lays the groundwork for advancing apMNKQ2 toward clinical evaluation in patients with PDAC, a tumor in need of more effective therapeutic options.

## Supplementary Information

Supplementary Material 1

## Data Availability

FASTQ RNAseq data files generated have been deposited in Sequence Read Archive (SRA) of the in the European Nucleotide Archive (ENA) with accession number ID: PRJEB94044. All data related to the results are available as part of the article. Additional information will be available on request.

## Abbreviations

PDAC: Pancreatic ductal adenocarcinoma
CSC: Cancer stem cell
MNK1: MAP kinase-interacting kinase 1
eIF4E: Eukaryotic translation initiation factor 4E
apMNKQ2: Anti-MNK1 DNA aptamer
PDX: Patient-derived xenograft
EMT: Epithelial-to-mesenchymal transition
CD133: Prominin-1 (a CSC marker)
CXCR4: C-X-C chemokine receptor type 4
GSEA: Gene set enrichment analysis
MCL1: Myeloid cell leukemia 1
XIAP: X-linked inhibitor of apoptosis protein
PK: Pharmacokinetics
ADME: Absorption, distribution, metabolism, and excretion
HPLC: High-performance liquid chromatography
PBS: Phosphate-buffered saline
RT-qPCR: Reverse transcription quantitative polymerase chain reaction
FBS: Fetal bovine serum
MTT: (3-(4,5-Dimethylthiazol-2-yl)-2,5-diphenyltetrazolium bromide) assay
DAPI: 4′,6-Diamidino-2-phenylindole
IC50: Half maximal inhibitory concentration
ANOVA: Analysis of variance
CD31: Platelet endothelial cell adhesion molecule
IVIS: In vivo imaging system
BM: Bone marrow
SC: Subcutaneous
IV: Intravenous
HEPES: 4-(2-Hydroxyethyl)-1-piperazineethanesulfonic acid
