# Supplementary material for: Aptamer-based inhibition of MNK1 reduces pancreatic ductal adenocarcinoma growth by targeting cancer stem cells
Source: J Biomed Sci. 2026 Jul 6;33:72. doi: 10.1186/s12929-026-01275-6 (PMC13335277; doi:10.1186/s12929-026-01275-6)
Supplement: Supplementary file 1 — Supplementary Material 1 [file 12929_2026_1275_MOESM1_ESM.pdf]

Aptamer-based inhibition of MNK1 reduces pancreatic ductal adenocarcinoma growth by targeting cancer stem cells

Alberto Pérez-Ruiz[1,†], Laura Ruiz-Cañas[2,3,4,†], Sandra Batres[2,3,†], Raquel Ferreras-Martín[1], Celia Pinto-Díez[1,5], Balbino Yagüe[2,3], Maria Isabel Pérez-Morgado[1], Silvia Sacristán[1], Ignacio Ruz Caracuel[6,7,8], Sonia Camaño Páez[4], Isabel Sanchez-Perez[2,3,9,10], Víctor M. González[1], Sonia Alcalá[1,2,3,*], María Elena Martín[1,*], Bruno Sainz, Jr.[2,3,*]

[1]Aptamer Group, Department of Biochemistry-Research, Instituto Ramón y Cajal de Investigación Sanitaria (IRYCIS)-Hospital Universitario Ramón y Cajal, 28034, Madrid, Spain
[2]Department of Cancer, Biomedical Research Institute (IIBm) Sols-Morreale CSIC-UAM, Madrid, 28029, Spain
[3]Biomarkers and Personalized Approach to Cancer Group (BIOPAC), Area 3 Cancer, IRYCIS, Madrid, 28034, Spain
[4]Biobank Hospital Ramón y Cajal-IRYCIS, Spanish National Biobanks Network (ISCIII Biobank Register No. B.0000678), IRYCIS, Madrid, 28034, Spain
[5]Aptus Biotech SL, Av. Cardenal Herrera Oria 298, Madrid, 28035, Spain
[6]Pathology Department, Hospital Universitario Ramón y Cajal, Madrid, 28034, Spain
[7]Molecular Pathology of Cancer Group, (IRYCIS), Area 3 Cancer, IRYCIS, Madrid, 28034, Spain
[8]Centro de Investigación Biomédica en Red, Área Cáncer, CIBERONC, Instituto de Salud Carlos III, Madrid, 28029, Spain
[9]Universidad Autónoma de Madrid, School of Medicine, Department of Biochemistry, Madrid, 28029, Spain
[10]Centro de Investigación Biomédica en Red, Área Enfermedades Raras, CIBERER, Instituto de Salud Carlos III, Madrid, 28029, Spain

[*]To whom correspondence should be addressed. Email: bsainz@iib.uam.es. Correspondence may also be addressed to salcala@ext.iib.uam.es and m.elena.martin@hrc.es.

[†]Alberto Pérez-Ruiz, Laura Ruiz-Cañas and Sandra Batres contributed equally to this work

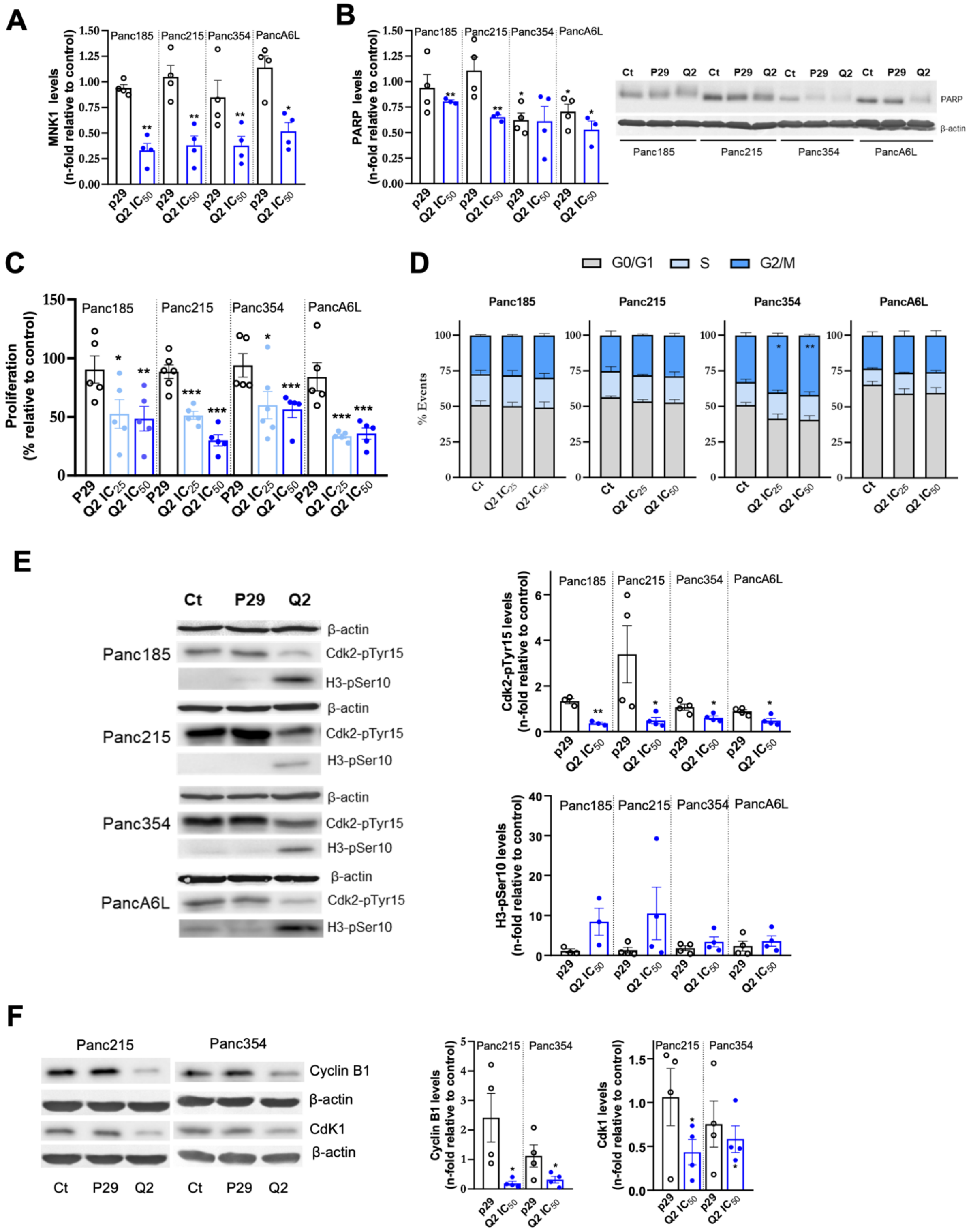

Figure S1. Effect of apMNKQ2 on PDAC cell cycle. A) Quantification of MNK1 protein levels. A representative Western blot is shown in Figure 1B. The graph represents the mean ±

SEM of 3-4 independent experiments. B) Quantification of total PARP protein levels. The graph represents the mean ± SEM of 3-4 independent experiments. A representative Western blot is shown on the right. C) Giemsa-based proliferation assay of four PDX-derived cells transfected with p29 (control) or apMNKQ2 at IC25 and IC50 concentration. The graph represents the means ± SEM of 5-6 independent experiments. D) Cell cycle distribution analysis by flow cytometry. Cells were transfected with apMNKQ2 at IC25 and IC50 concentrations and 24h later stained with FxCycle™ Violet Stain for cytometer analysis. Graphs represent the means ± SEM of 4 independent experiments. E) Cell cycle distribution analysis based on molecular markers for the G1/S and M phases. Western blot with Cell Cycle (pCdk/pHH3/Actin) WB Cocktail of cells transfected with p29 (control) or apMNKQ2 aptamer at IC50 concentration for 24 h. The graphs represent the means ± SEM of 3-4 independent experiments. Representative blots are shown on the left of the figure. F) WB analysis of Cyclin B1 and CDK1 protein levels in PDX-derived cell lines 24 hours after p29 (control) or apMNKQ2 aptamer transfection at IC50 concentration. β-actin is included as a loading control. The graphs represent the means ± SEM of 4 independent experiments. Representative blots are shown on the left of the figure. *$p < 0.05$; **$p<0.01$; ***$p<0.001$ relative to control; as determined by one-sample t test.

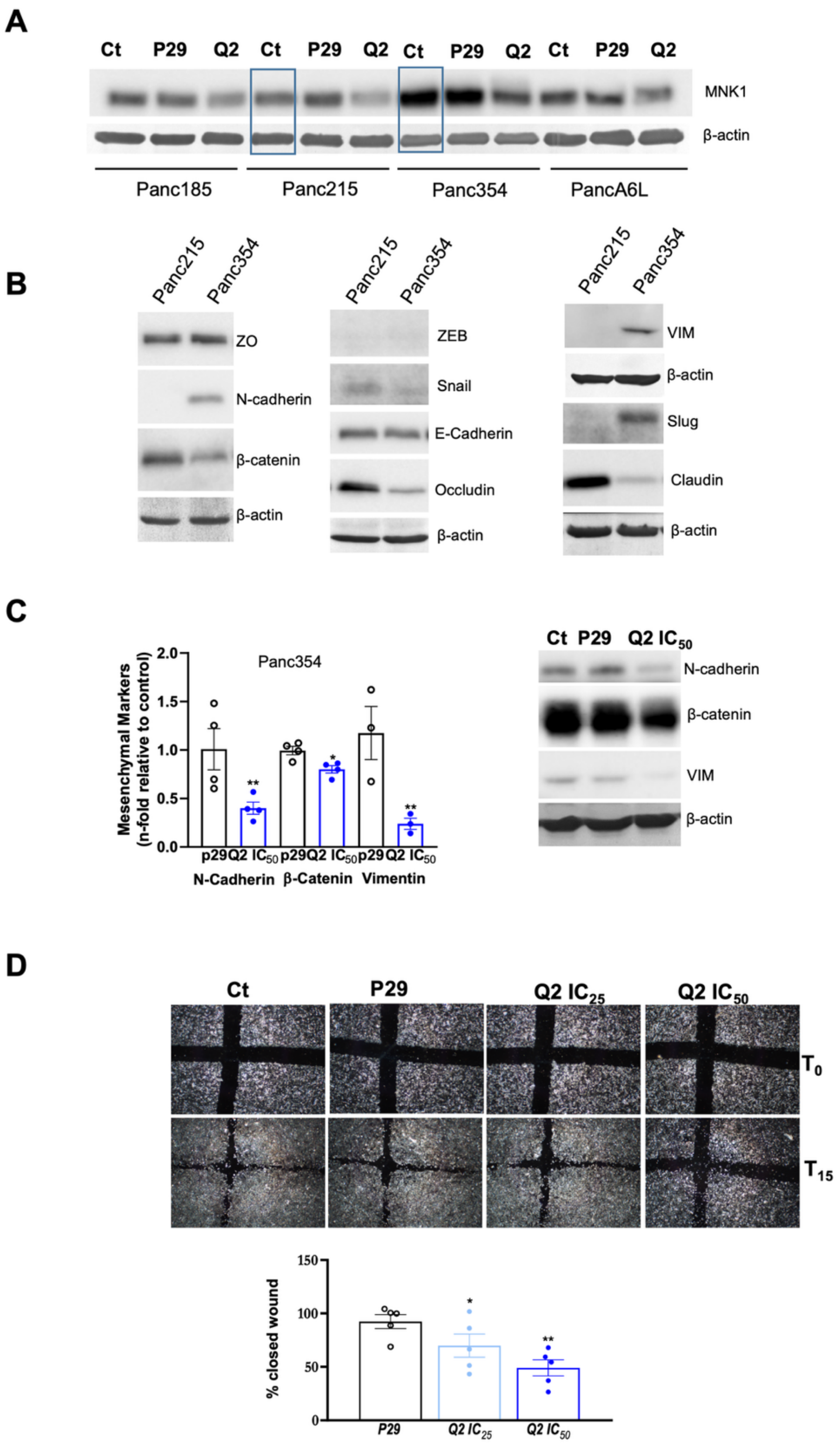

Figure S2. Differential expression of EMT/cell membrane proteins and migration capacity. A) Representative WB of MNK1 protein levels in the four PDX cell lines not transfected or transfected with P29 or apMNKQ2 (Q2). Square grids highlight the different MNK1 protein expression levels between Panc215 and Panc354. B) Representative WB of EMT and cell membrane protein levels in Panc215 and Panc354 cell lines. β-actin is included as a loading control. C) WB analysis of mesenchymal markers in Panc354 cell line 24 h after

P29 (control) or apMNKQ2 (Q2) aptamer transfection at IC50 concentration. β-actin is included as a loading control. The graph represents the means ± SEM of 3-4 independent experiments. Representative blots are shown on the right. D) Panc354 cells untransfected (control; Ct) or transfected with p29 or apMNKQ2 (Q2) at IC25 or IC50 concentrations were treated with mitomycin and a wound was induced with a pipette tip. Representative photomicrographs of initial and final wounds are shown for 0 (T_0) and 15 (T_{15}) hours post wound initiation, respectively. The percentage of closed wound was determined by dividing the size of the wound at the end of the experiment (T_{15}) by the size of the wound at the initiation of the assay (T_0). The graphs represent the means ± SEM of 5 independent experiments. *$p < 0.05$; **$p<0.01$ relative to control; as determined by one-sample t test.

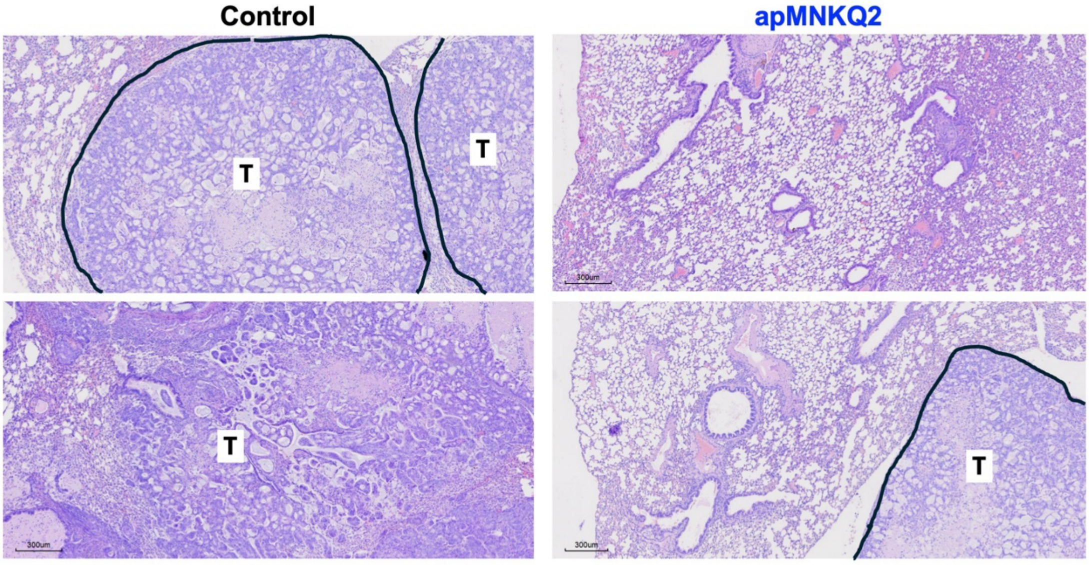

Figure S3. apMNKQ2 inhibits the metastatic capacity of PDAC cells. Representative micrographs of H&E-stained lungs from Figure 3D. T = tumor.

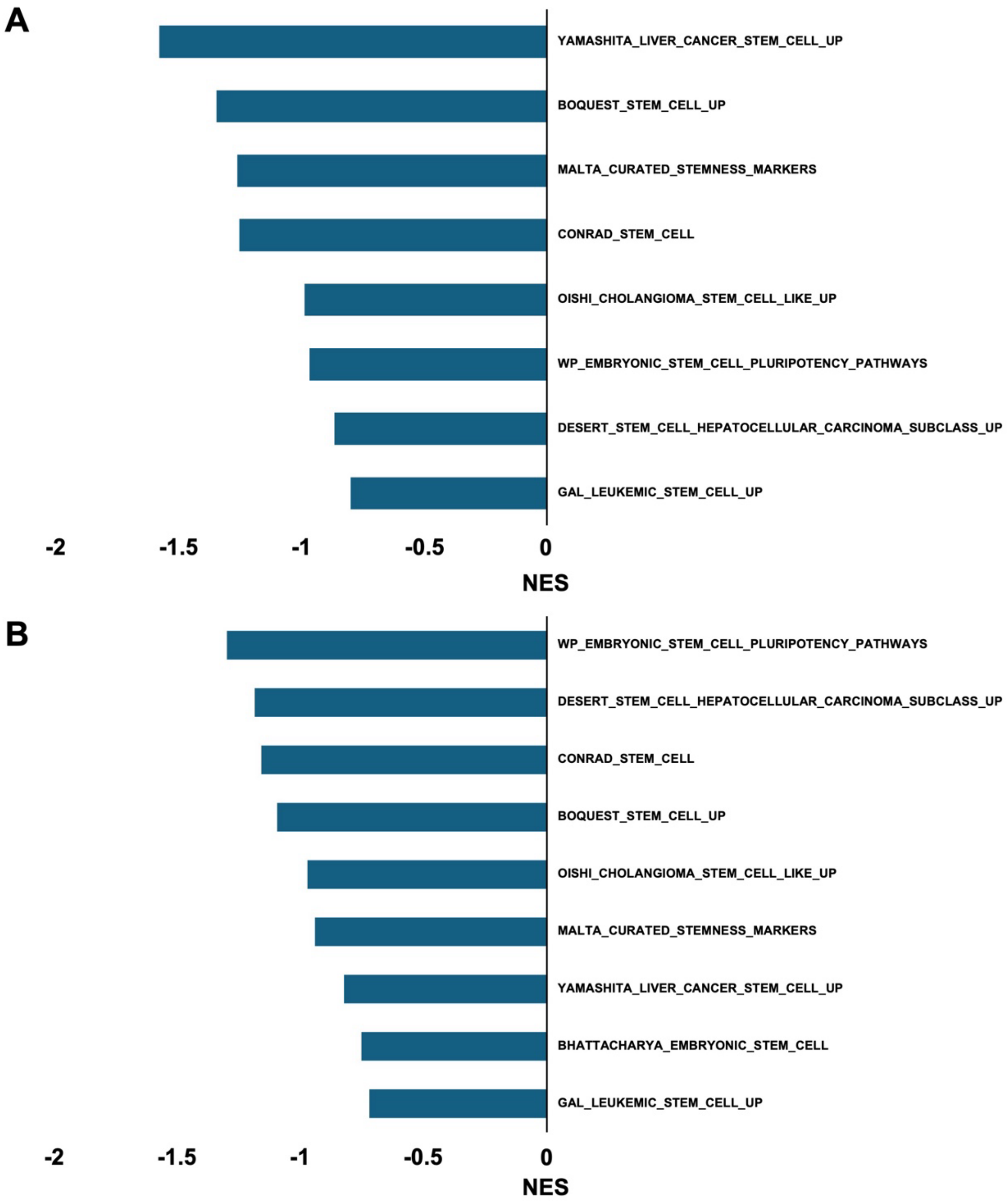

Figure S4. Down-regulation of stem-associated pathways in apMNKQ2-treated PDX Panc cells. A-B) Summary of GSEA normalized enrichment scores (NES) of the indicated stem-related pathways in apMNKQ2- versus control-transfected Panc215 (A) or Panc354 (B) cells.

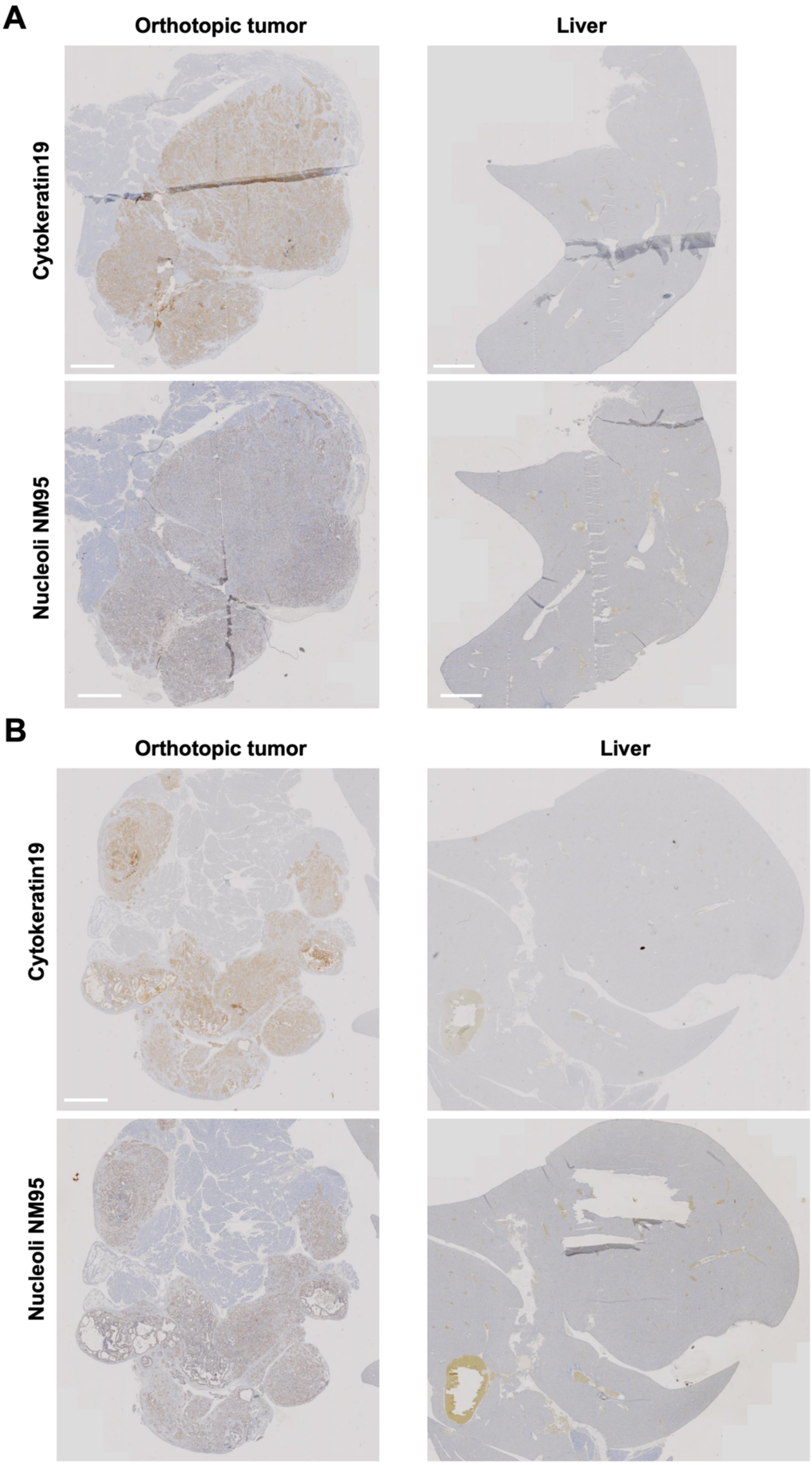

Figure S5: CK19 and Nucleoli NM95 staining in Panc215 orthotopic tumors and livers. IHC of Cytokeratin19 or Nucleoli NM95 (a human-specific nuclear probe) in orthotopic tumors (left) and livers (right) from mice 28 days post injection in the pancreas with control (A) or apMNKQ2-transfecetd (B) Panc215 cells.

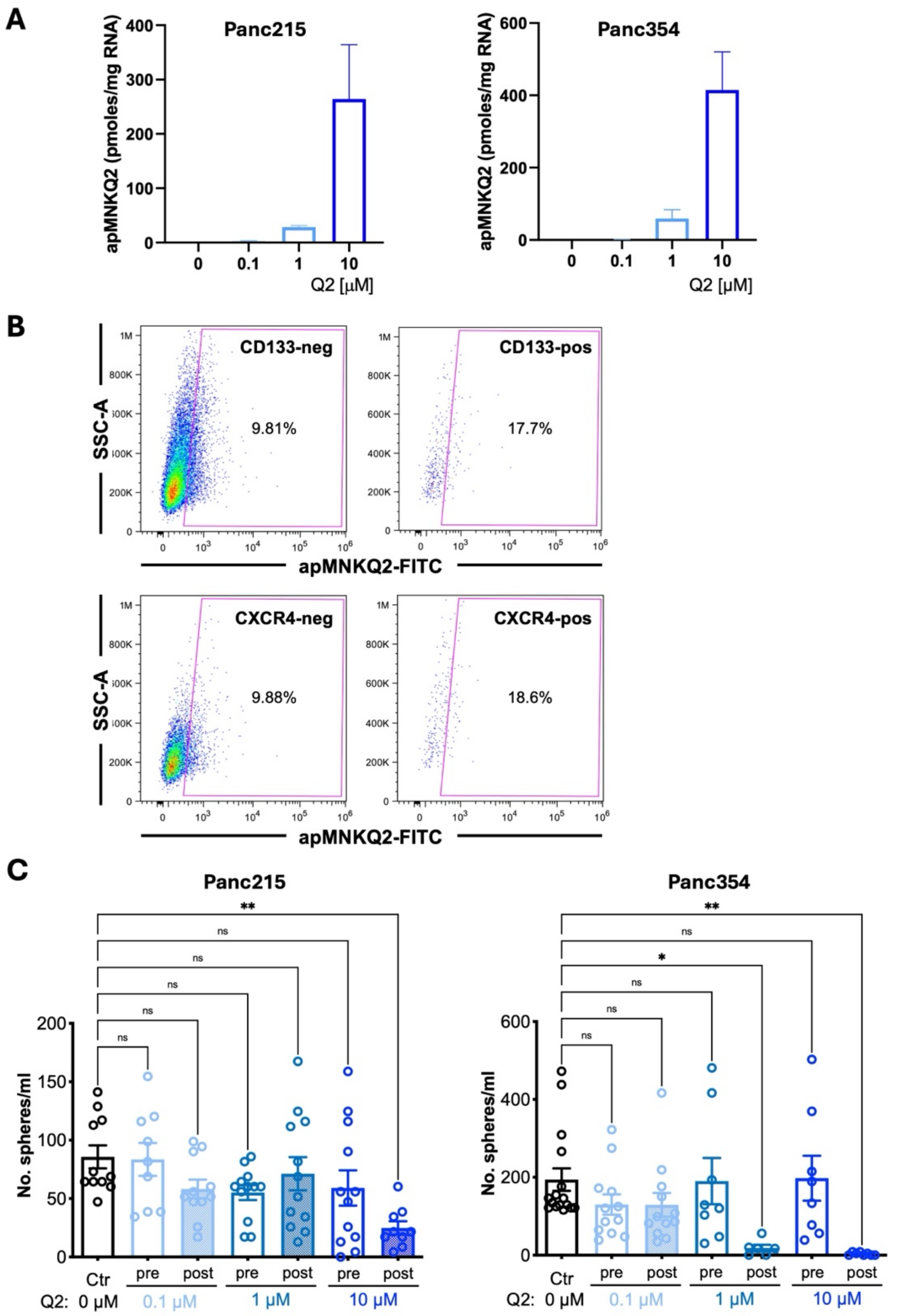

Figure S6. Free apMNKQ2 efficiently targets PDAC CSCs. A) Mean pmol/mg of RNA of apMNKQ2 ± SEM, determined by qPCR, 24 h post control-(0 µM) or apMNKQ2- (0.1, 1 or 10 µM) treatment of Panc215 or Panc354 cells. B) Representative flow cytometric plots of apMNKQ2-FITC accumulation in CD133 and CXCR4 negative (neg) and positive (pos) cells. C) Mean number (no.) of spheres/ml ± SEM in control (Ctr)- or pre and post apMNKQ2-treated (0.1, 1 or 10 µM) cells 5 days post 2nd generation sphere initiation. * $p < 0.05$, ** $p < 0.01$, ns= not-significant; by One-way ANOVA with Dunnett's multiple comparison test.

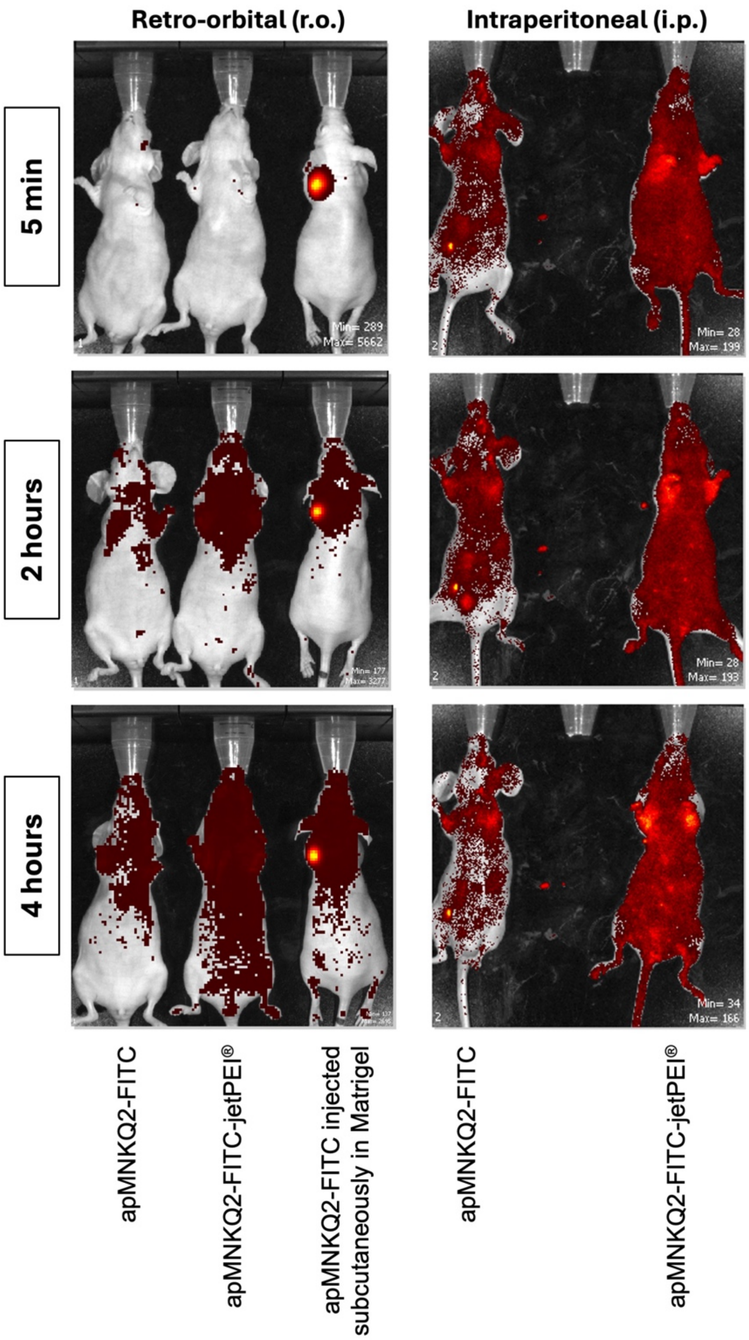

Figure S7. Distribution of apMNKQ2-FITC in real time. IVIS images of mice following a single dose of 1.7 mg/Kg of apMNKQ2-FITC administered retro-orbitally (r.o.) or intraperitoneally (i.p.) to CD1 Swiss mice with jetPEI® or without (i.e., selection buffer). Images were obtained 5 min, 2 h or 4 h post injection. As a positive control, a mouse was subcutaneously injected with apMNKQ2-FITC embedded in Matrigel.

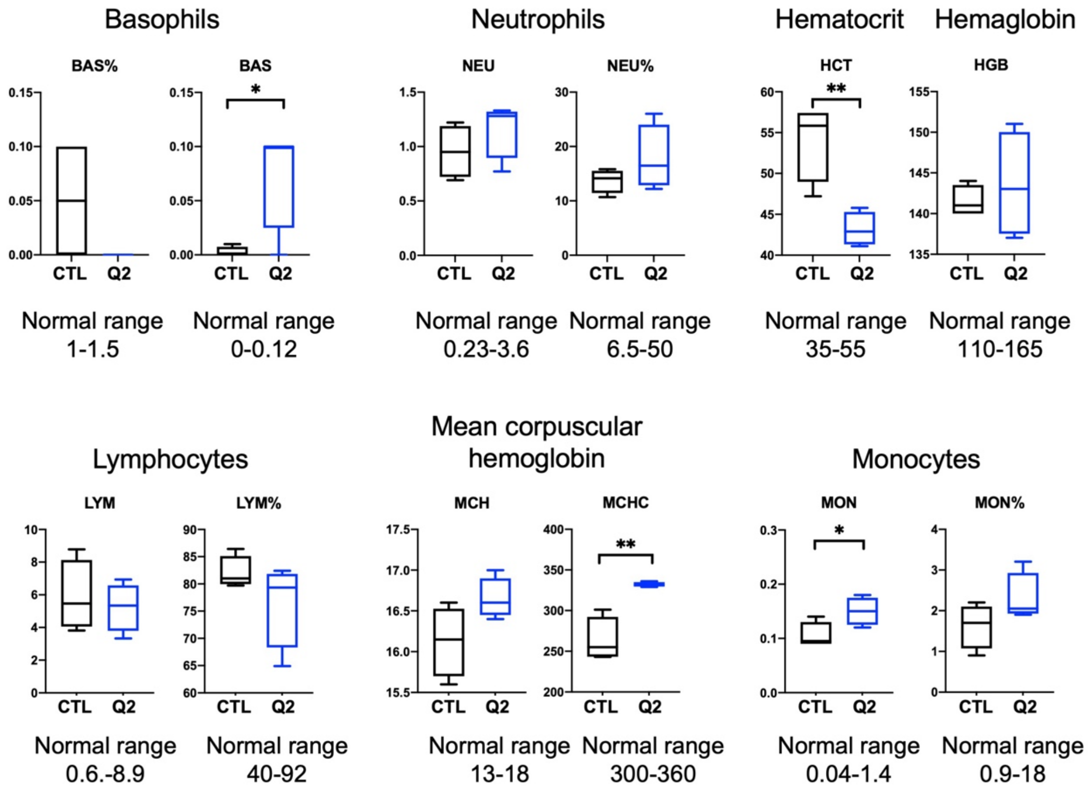

Figure S8. Hematocrit analysis of apMNKQ2 in vivo. Analysis of the indicated hematocrit parameters in mice treated with 0 (CTL) or 400 mg/Kg of free apMNKQ2 (Q2) for 30 consecutive days. Box plots indicate the mean ± SEM of the levels determined. Established normal range parameters are indicated. (n=5 mice per group). *p < 0.05; **p<0.01 relative to control; as determined by one-sample t test.

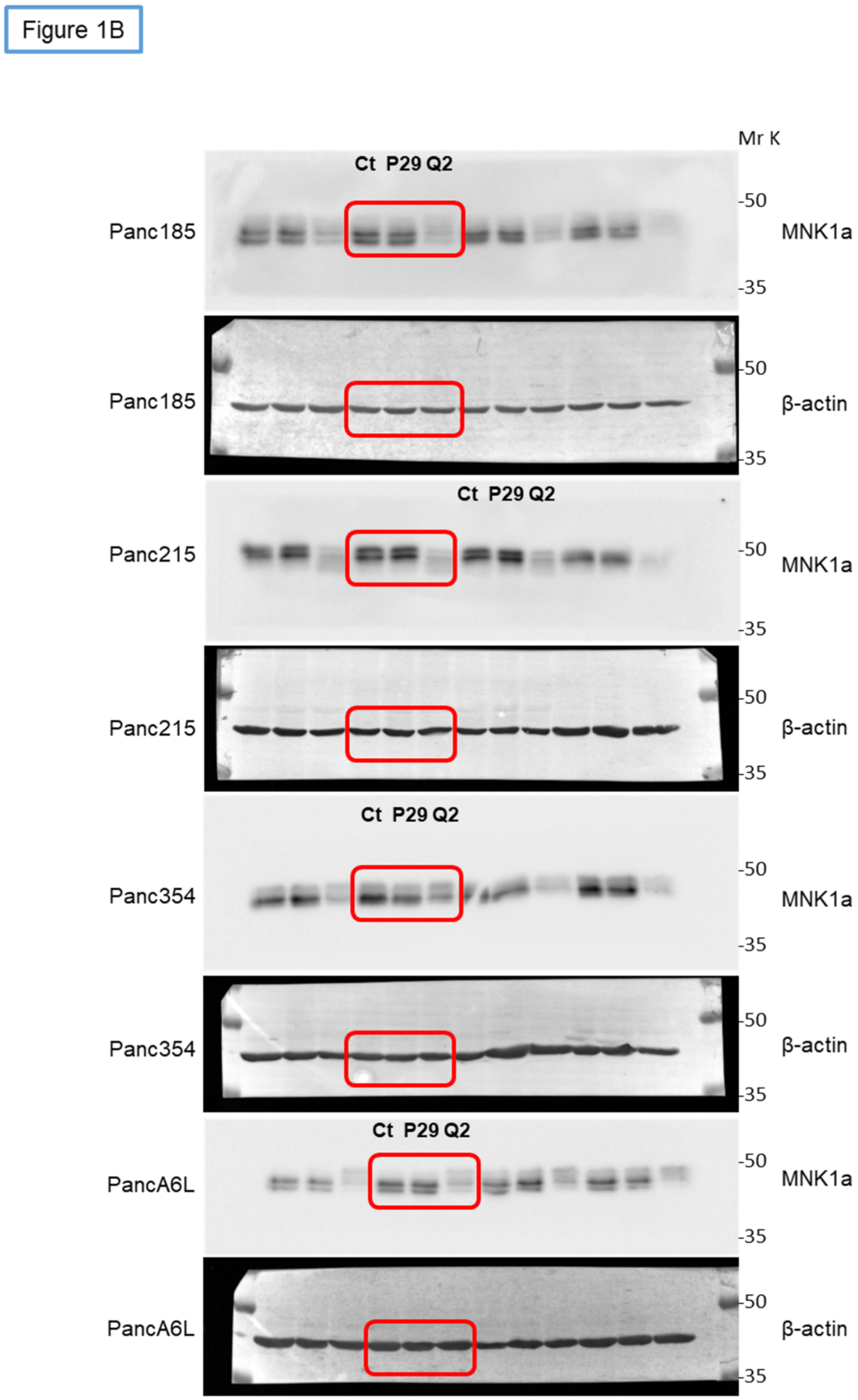

108

109 **Figure S9: Uncropped original Western Blot images for Figure 1B**

Figure 1E

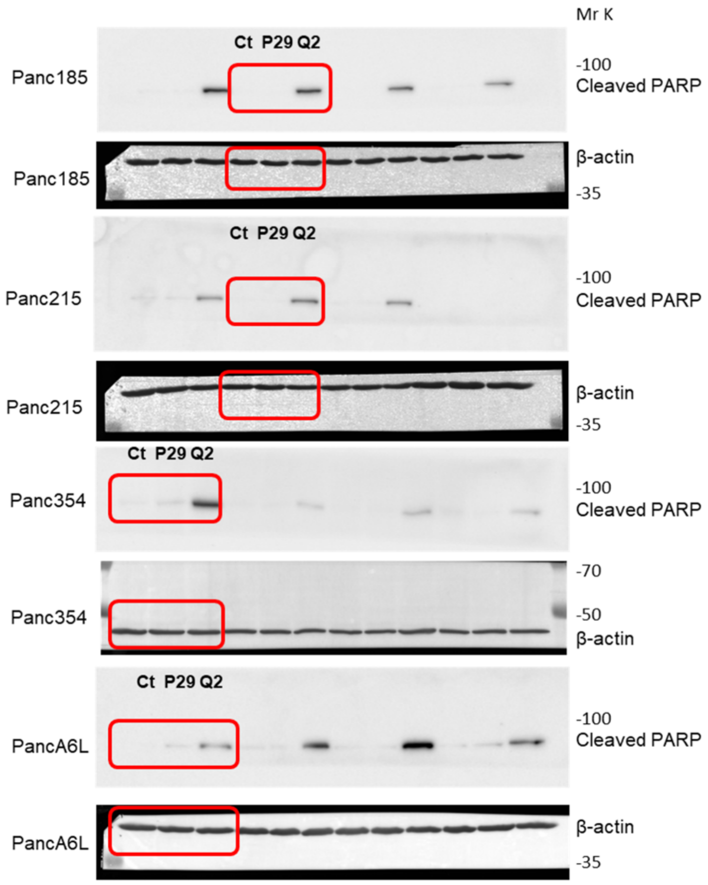

Figure 1F

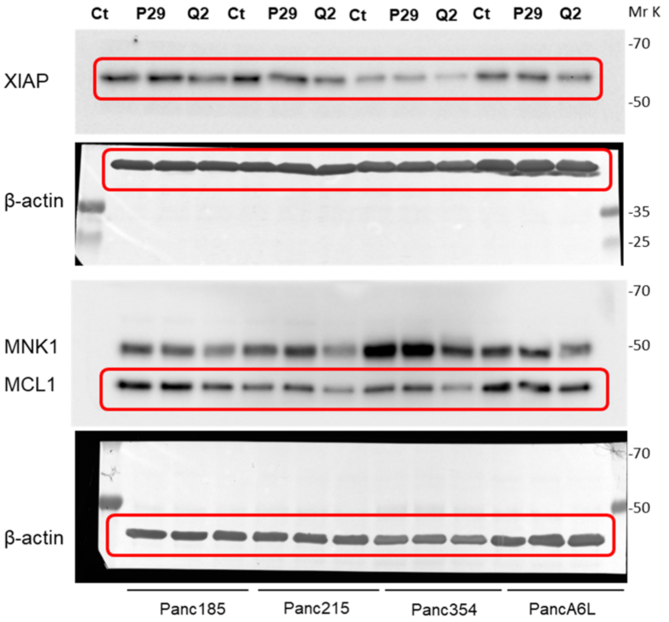

Figure S10: Uncropped original Western Blot images for Figures 1E and 1F

Figure 2D

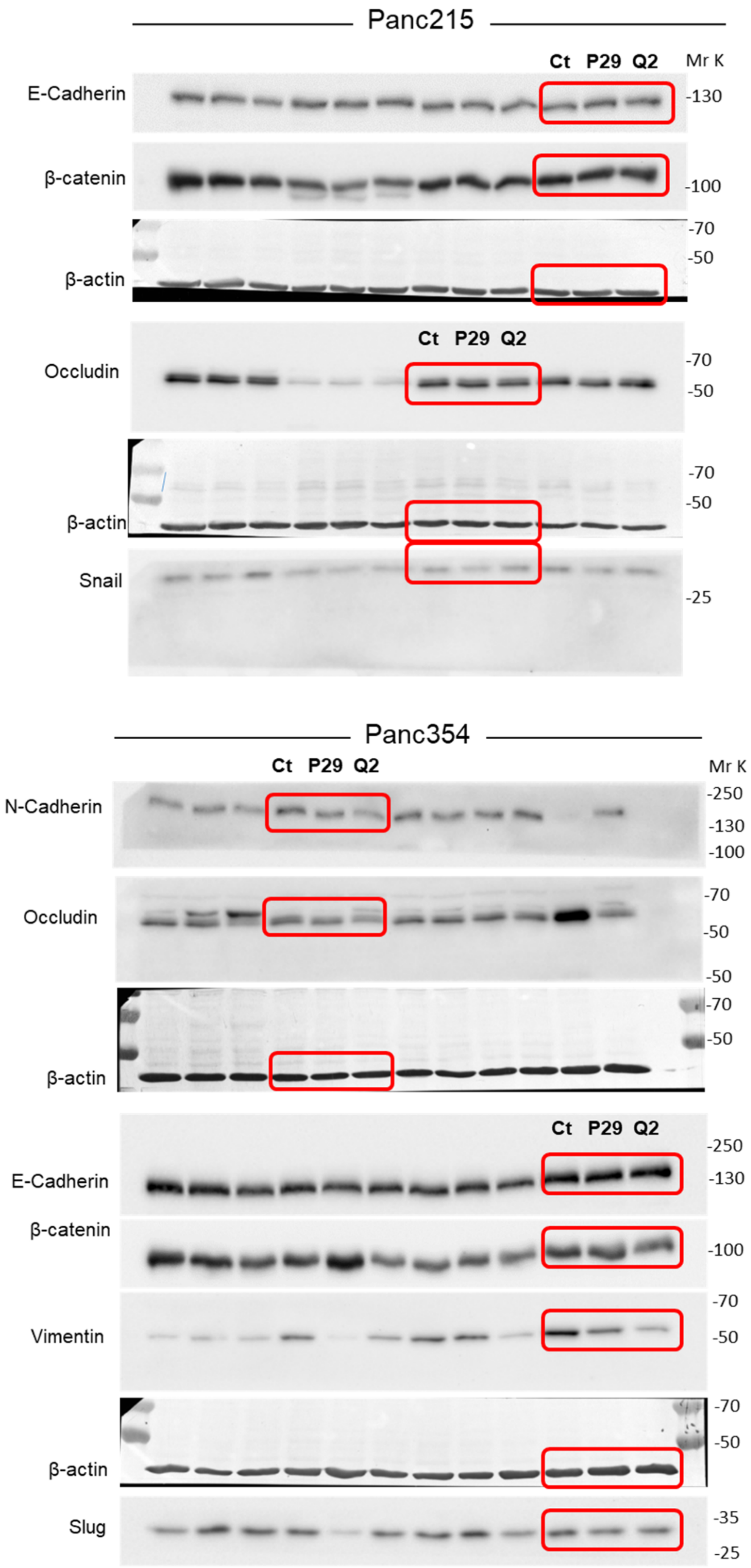

Figure S11: Uncropped original Western Blot images for Figure 2D

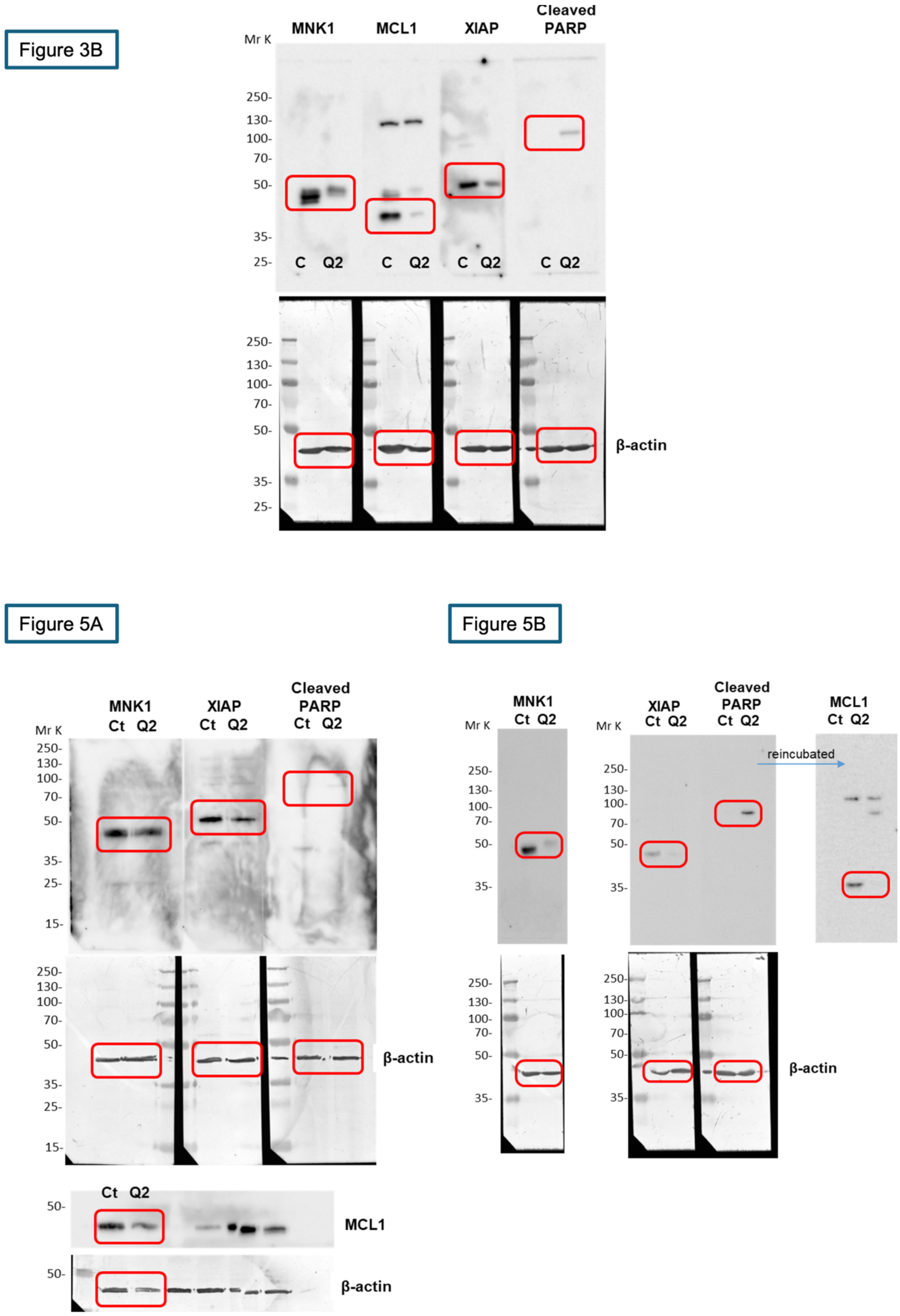

Figure S12: Uncropped original Western Blot images for Figures 3B, 5A and 5B

Figure 6B

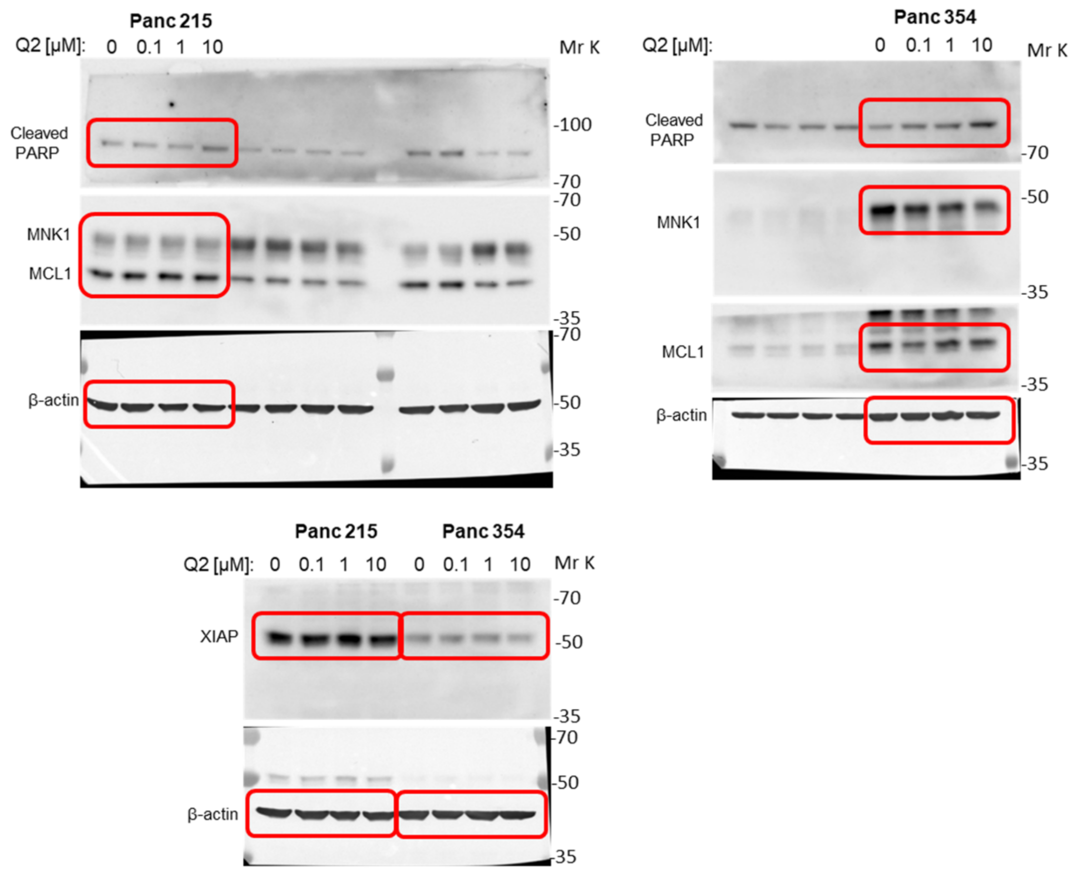

Figure S13: Uncropped original Western Blot images for Figure 6B

Supplementary Table S1: Antibodies

Antibody	Source	Identifier	Dilution
β-Actin	Sigma	A5441	1:5000 for WB
β-Catenin (D10A8)	Cell Signaling	8480	1:2000 for WB
Claudin-1 (D5H1D)	Cell Signaling	13255	1:2000 for WB
CD133-APC	Miltenyi	130-111-756	1:50 for FC
CXCR4-PE	Miltenyi	130-117-354	1:50 for FC
CD24-PE	BD	555428	1:5 for FC
Cell Cycle (pCdk/pHH3/Actin) WB Cocktail	Abcam	ab136810	1:1000 for WB
Cleaved PARP (Asp214)	Cell Signaling	9541	1:1000 for WB
CDK1	SantaCruz Biotechnology	sc-54	1:1000 for WB
Cyclin B1	SantaCruz Biotechnology	Sc-166757	1:1000 for WB
E-cadherin (24E10)	Cell Signaling	3195	1:1000 for WB
MCL-1	Cell Signaling	39224	1:1000 for WB
MNK1(C4C1)	Cell Signaling	2195	1:1000 for WB
N-Cadherin (D4R1H)	Cell Signaling	13116	1:1000 for WB
Occludin	GENETEX	GTX114949	1:1000 for WB
Slug (C19G7)	Cell Signaling	9585	1:1000 for WB
Snail (C15D3)	Cell Signaling	3879	1:1000 for WB
PARP	SantaCruzBiotechnology	Sc8007	1:2000 for WB
PCNA (PC10)	SantaCruzBiotechnology	Sc-56	1:1000 For IHC
Vimentin (D21H3)	Cell Signaling	5741	1:1000 for WB
XIAP	BD Biosciences	610716	1:1000 for WB
XIAP	Novus Biologicals	NBP2-20918	1:1000 For IHC
ZEB1	GENETEX	GTX114541	1:1000 for WB
CK19	DAKO-Agilent	IR0615	Pre-diluted For IHC
Nucleoli NM95	Abacm	ab197710	1:18,000 For IHC
ZO-1 (D7D12)	Cell Signaling	8193	1:1000 for WB

Supplementary Table 2: Gene signatures

Name	No. genes	Description	Species	Reference
YAMASHITA_LIVER_CANCER _STEM_CELL_UP	47	Genes up-regulated in hepatocellular carcinoma (HCC) cells with hepatic stem cell properties	Homo sapiens	[1]
BOQUEST_STEM_CELL_UP	261	Genes up-regulated in freshly isolated CD31- (stromal stem cells from adipose tissue) versus the CD31+ (non-stem) counterparts	Homo sapiens	[2]
MALTA_CURATED_STEMNESS _MARKERS	21	Literature curated collection of genes marking normal and cancer stem cells	Homo sapiens	[3]
CONRAD_STEM_CELL	39	Supplementary Table 2. Genelist comparing microarray expression profiles of spermatogonial cells, haGSCs and hES (H1) cells. Examples of expression rates of different hES cell enriched and germ cell specific genes, surface markers for germ cell selection and signal transduction in all three cell types (spermatogonial cells = SC)	Homo sapiens	[4]
OISHI_CHOLANGIOMA_STEM _CELL_LIKE_UP	349	Genes over-expressed in stem cell-like cholangiocellular carcinoma	Homo sapiens	[5]
WP_EMBRYONIC_STEM_CELL _PLURIPOTENCY_PATH WAYS	117	Embryonic stem cell pluripotency pathways	Homo sapiens	GSEA C2:Curated CP:Canonical Pathways CP:WikiPathways
DESERT_STEM_CELL_ HEPATOCELLULAR_ CARCINOMA_SUBCLASS_UP	242	Genes up-regulated in the stem cell-type subclass of hepatocellular carcinomas.	Homo sapiens	[6]
GAL_LEUKEMIC_STEM_ CELL_UP	130	Genes up-regulated in leukemic stem cells (LSC), defined as CD34+CD38- [GeneID=947;952] cells from AML (acute myeloid leukemia patients) compared to the CD34+CD38+ cells.	Homo sapiens	[7]
BHATTACHARYA_EMBRYONIC_ STEM_CELL	84	The 'stemnes' signature: genes up-regulated and common to 6 human embryonic stem cell lines tested.	Homo sapiens	[8]

Supplementary References

[1] Yamashita T, Ji J, Budhu A, et al. EpCAM-positive hepatocellular carcinoma cells are tumor-initiating cells with stem/progenitor cell features. Gastroenterology. 2009;136:1012-1024.
[2] Boquest AC, Shahdadfar A, Fronsdal K, et al. Isolation and transcription profiling of purified uncultured human stromal stem cells: alteration of gene expression after in vitro cell culture. Molecular biology of the cell. 2005;16:1131-1141.
[3] Malta TM, Sokolov A, Gentles AJ, et al. Machine Learning Identifies Stemness Features Associated with Oncogenic Dedifferentiation. Cell. 2018;173:338-354 e315.
[4] Conrad S, Renninger M, Hennenlotter J, et al. Generation of pluripotent stem cells from adult human testis. Nature. 2008;456:344-349.
[5] Villanueva A, Hoshida Y, Battiston C, et al. Combining clinical, pathology, and gene expression data to predict recurrence of hepatocellular carcinoma. Gastroenterology. 2011;140:1501-1512 e1502.
[6] Desert R, Rohart F, Canal F, et al. Human hepatocellular carcinomas with a periportal phenotype have the lowest potential for early recurrence after curative resection. Hepatology. 2017;66:1502-1518.
[7] Gal H, Amariglio N, Trakhtenbrot L, et al. Gene expression profiles of AML derived stem cells; similarity to hematopoietic stem cells. Leukemia. 2006;20:2147-2154.
[8] Bhattacharya B, Miura T, Brandenberger R, et al. Gene expression in human embryonic stem cell lines: unique molecular signature. Blood. 2004;103:2956-2964.
